# Preserving for the future the — once widespread but now vanishing — knowledge on traditional pig grazing in forests and marshes (Sava-Bosut floodplain, Serbia)

**DOI:** 10.1186/s13002-021-00482-9

**Published:** 2021-10-09

**Authors:** Zsolt Molnár, Klára Szabados, Alen Kiš, Jelena Marinkov, László Demeter, Marianna Biró, Kinga Öllerer, Krisztián Katona, Marko Đapić, Ranko Perić, Viktor Ulicsni, Dániel Babai

**Affiliations:** 1 Centre for Ecological Research, Institute of Ecology and Botany, Vácrátót, 2163 Hungary; 2 Institute for Nature Conservation of Vojvodina Province, Novi Sad, 21101 Serbia; 3 MTA-DE ‘Lendület’ Evolutionary Phylogenomics Research Group, Hungarian Academy of Sciences, Debrecen, 4032 Hungary; 4 Institute of Biology Bucharest, Romanian Academy, Bucharest, 060031 Romania; 5 Institute for Wildlife Management and Nature Conservation, Hungarian University of Agriculture and Life Sciences, Gödöllő, 2100 Hungary; 6 Research Centre for the Humanities, Institute of Ethnology, Budapest, 1097 Hungary

**Keywords:** Knowledge generation, Cultural heritage, Conservation management, Sustainable use of natural resources, Foraging, *Sus scrofa domestica*, Wild boar

## Abstract

**Background:**

Traditional knowledge is key for sustainability, but it is rapidly disappearing. Pig keeping in forests and marshes is an ancient, once widespread, now vanishing practice, with a major economic and ecological potential. The knowledge of pig keepers and the foraging activity of pigs are hardly documented.

**Methods:**

We studied the knowledge of traditional pig keepers (*svinjar*s) on wild plants and pig foraging on the Sava-Bosut forest-marsh complex in Serbia. We conducted picture-based interviews about 234 locally common and/or salient plant species, and participatory fieldwork (11 days) and visual observation (21 days) on pig foraging.

**Results:**

181 wild plant species were known by *svinjar*s and 106 taxa were consumed by pigs. *Svinjar*s knew well and could name most regularly foraged species. 98 species were reported by *svinjar*s as foraged and 56 as not eaten. 28 species were observed by the authors as eaten regularly, while 21 were nibbled and 17 avoided. Contradictory information on foraging was rare both among *svinjar*s (8 species) and between *svinjar*s and researchers (7 species); several of these species were rare. Leaves of 92, fruits or seeds of 21 and ‘roots’ of 20 species were reported or observed as eaten, usually with high seasonality. *Svinjar*s were overall observant, but knew little about some less salient species (e.g. *Veronica*, *Circaea*). The most common forages (reported and/or observed) were fruits (*Quercus*, fleshy fruits), grasses (*Agrostis*, *Glyceria*), herbs (*Ranunculus ficaria*, *Circaea*), nutritious ‘roots’ (*Carex* spp., *Iris*), young shrub leaves (*Crataegus*, *Carpinus*) and ‘tame’ plants growing in the sun (*Persicaria dubia*, *Erigeron annuus*). Traditional, now extinct pig breeds were reported as less selective and more ‘knowledgeable’ about plants, as they received less additional fodder. *Svinjar*s learnt their knowledge since childhood, from community members, but long-term personal observations and everyday encounters with pigs were also important sources of knowledge.

**Conclusions:**

A deeper understanding of pig foraging could contribute to using pigs in nature conservation management, resource management and organic farming, and to a better understanding of wild boar foraging. The knowledge of *svinjar*s is a disappearing intangible cultural heritage of European importance. Knowledge holders deserve recognition, and legal and financial support to continue this tradition.

## Background

Extensive traditional livestock husbandry systems often produce high-quality food, create and maintain new species-rich habitats (e.g. hay meadows, wood-pastures), and manage ecosystems for biodiversity conservation [1, 2]. These traditional land-use practices and the related traditional ecological knowledge, are vital for sustainability but they are rapidly disappearing [3]. Knowledge on cattle and sheep grazing is relatively well documented [4, 5] but extensive traditional pig grazing is rarely studied [6].

Keeping pigs in marshes and forests using extensive breeds has been a widespread practice in Europe for millennia [7–9]. Its importance is also indicated by the fact that in the past, forests in Europe were often valued (measured) based on how many pigs they could feed [7, 10–12]. Extensive pig keeping (especially masting) has been well documented since medieval times (e.g. [13, 14]), and even more so since the nineteenth century [12, 15–22]. Studies on recent extensive pig grazing, however, are scarce [6, 7, 23–25], and most often document the dehesa system with Iberian pigs kept in wood-pastures and dry grasslands (e.g. [26–28]). There is a huge knowledge gap regarding how free-ranging domestic pigs forage in semi-natural areas (forests, grasslands, marshes) and what ecological knowledge pig keepers possess (need) in such areas.

Today the practice of keeping pigs in closed forests and marshes is almost extinct in Europe, although a revival of the practice is advocated in nature conservation management and organic farming [6, 7, 14, 24, 29–33]. Allowing pigs to range free in semi-natural habitats has positive impacts on the health of the animals and the quality of their meat [34–37] is less resource-intensive (lower time, energy and feed costs; [31]), and is more environmentally friendly [38, 39]. The practice can even benefit red-listed protected species, e.g. marsh plants and birds [6, 40, 41].

European forests and wetlands have been radically transformed over the last centuries [42], traditional land-use practices have ceased [22, 33, 43], and multidimensionality of use has decreased [44, 45]. Prohibition of some traditional uses (e.g. forest grazing, pig grazing) has also contributed to the rapid decline and erosion of related traditional ecological knowledge [43], with severe biodiversity effects [46]. Due to the changing environment and lifestyles, innovations and novel adaptations, based on available knowledge and existing traditions, are needed for the successful continuation of extensive land-use practices (incl. pig keeping) and to further increase their benefits cf. [5, 33].

Ethnobiology—among other things—documents traditional knowledge and practices, the related values and worldviews, and the ways in which traditional knowledge is generated and transmitted [47, 48]. Ethnobotanical knowledge of medicinal and wild food plants is relatively well documented in Europe (e.g. [49, 50]) but major gaps still exist when it comes to knowledge related to pastoral practices, e.g. about forage plants [5, 51]. The practices and impacts of traditional livestock grazing on grasslands are also relatively well documented in Europe (e.g. [52–54]). Much less is known about extensive traditional forest and marsh grazing and the foraging behaviour of livestock in these habitats [33, 43, 46, 55], especially about pig grazing [6, 31].

Ethnobiologists understand their responsibility to document and archive traditional (folk) knowledge, and to support the continuation of traditional practices and ongoing adaptation and knowledge generation [3, 56, 57]. Documenting traditional knowledge can help knowledge transmission and revival programmes [58–60] and assist in developing culturally appropriate agricultural and conservation regulations and subsidies [61, 62].

We are not aware of any recent ethnobiological scientific study on the existing ecological knowledge of traditional pig keepers rearing pigs in semi-natural forests, grasslands and marshes in Europe. All studies are ethnographical or historical, and document historical knowledge and practices. For example, Rodríguez-Estévez et al. [63] list 133 wild and cultivated forage taxa that were traditionally collected and given to dehesa pigs in Spain, while Szabadfalvi [15] reviewed traditional pig keeping in Hungary.

The domestic pig (*Sus scrofa domestica* L.) belongs to the same species as the wild boar, and there is a rich literature on wild boar foraging on various plant species [64, 65]. Wild boar can cause serious damage, especially in crop fields, so studies focus more on agricultural areas and crops than on forests and marshes [66–68]. In detailed case studies conducted in semi-natural landscapes, 32–104 wild plant species were found to be foraged by wild boars [64, 69], the closest to our study area [70] but identification of foraged plant species is limited by the methodologies applied (stomach and faeces analysis) [65]. Observation-based studies on behaviour and foraging (including grazing and rooting) of wild boar are scarce [71–73]. Observational studies on free-ranging domestic pigs are also rare and done on non-traditionally kept pigs grazing in enclosures [23, 25]. Our study is the first on traditionally kept, free-ranging pigs foraging in semi-natural habitats.

The main objective of our study was to document the vanishing traditional ecological knowledge and practice of rearing pigs in closed (oak) forests and marshes in a semi-natural floodplain (the Sava-Bosut floodplain in Serbia). This knowledge was once widely possessed and applied across Europe (considering the former extent of the associated practice), but has almost completely disappeared. Now it is possessed only by 100 people at most, almost exclusively over 60 years of age, in some limited locations in Central Europe.

In this paper we studied (1) the traditional ecological knowledge of traditional forest pig keepers (called locally: *svinjar*) on wild plant species, specifically local folk names, local knowledge of pig–plant relationships (eat, love, avoid, toxic, medicinal etc.), other local uses and salient features of these plant species; and (2) how traditional knowledge of pig foraging behaviour and foraged plants was generated and transmitted in this community.

## Methods

### Study area

**Fig. 1:**
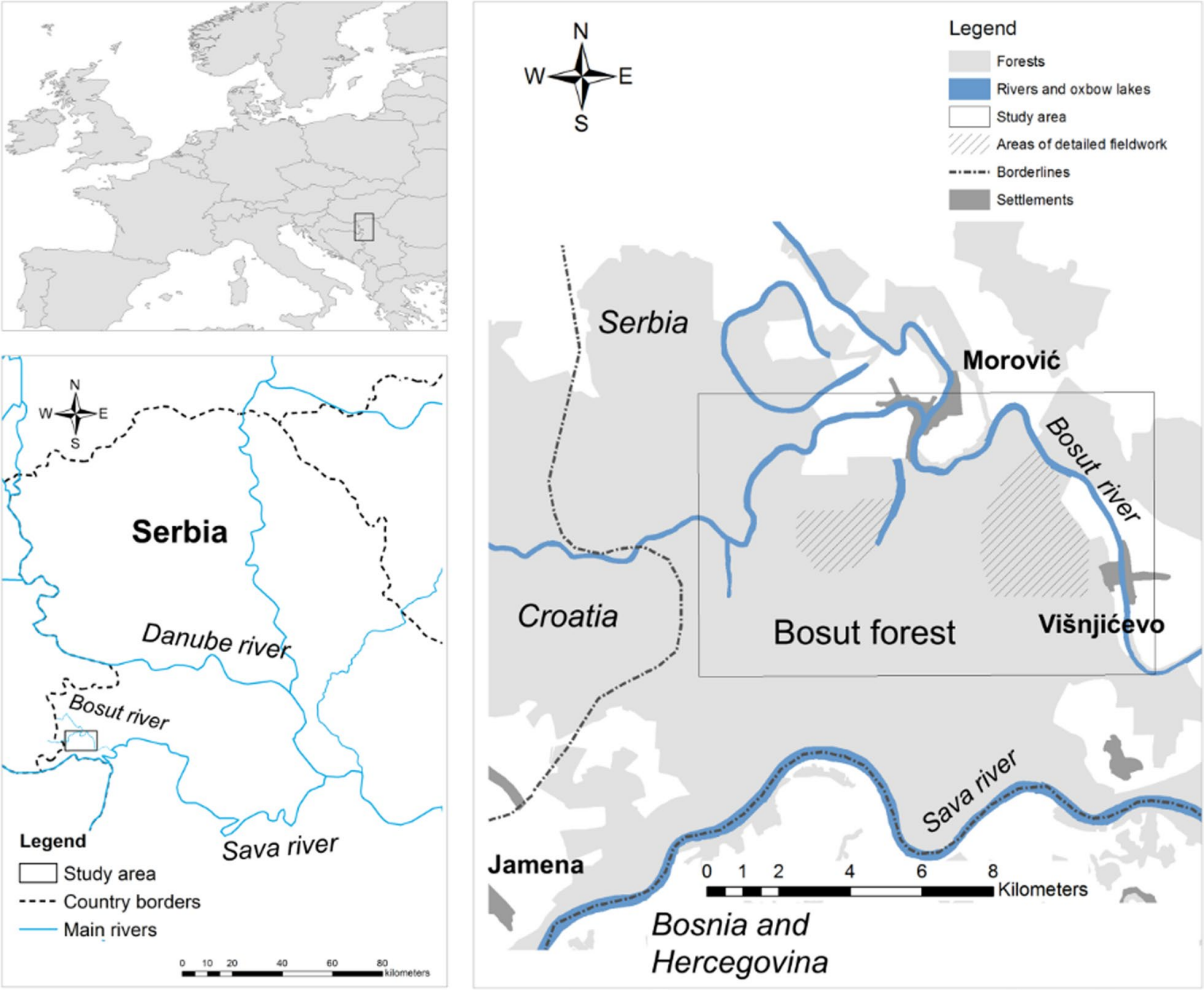
Study area on the floodplain of the Bosut and Sava rivers in Serbia, Central Europe. Striped polygons indicate areas visited during visual observation of pig foraging (source of country borders and main rivers: Natural Earth, [87]; source of forests, settlements, Bosut river and oxbow lakes: Corine Land Cover, [88])

Although forest grazing was common even in the second half of the twentieth century in the former Yugoslavia [74], nowadays the floodplains of the Sava, Bosut and Studva rivers are among the very last places where the foraging behaviour of free ranging pigs in closed forests and marshes can be studied in Central Europe [31, 32, 75]. We worked in the territory of the villages Morović and Višnjićevo (marginally in Jamena) close to the administrative borders between the Republic of Serbia, Bosnia & Herzegovina and the Republic of Croatia (Fig. 1).

The study area belongs to the famous Slavonian oak forest area [76]. The Bosut forest was for centuries part of the border zone between the Austro-Hungarian Monarchy and the Ottoman Empire. The high importance of large-scale pig keeping is documented at least from the seventeenth century [77]. Forest management favouring hardwood species (*Quercus*, *Fraxinus*, *Ulmus*, *Carpinus*) dates back to the formation of the Military Border [76]. With some technological changes, *Quercus robur* remained the major tree species [78] and was the cornerstone of pannage. Pig, cattle and sheep grazing was the main use of the forests till the end of the nineteenth century. Forests were managed by irregular selective cutting of valuable timber. With the increased interest of the European timber market in high quality oak timber in the mid of nineteenth century, forest management intensified and the importance of forest grazing decreased [77, 79]. The forests in the study area are now state owned.

The study area is dominated by forests with embedded floodplain marshes, and arable land around villages. The Bosut Forest (21,852 ha) is a local ecological hotspot, recognized as an Important Bird Area (RS007) and a core area of the National Ecological Network. The climate is subcontinental with 11 °C mean annual temperature and a yearly average precipitation of 700 mm [80]. The area was cut off from floods in the 1930s and became drier in the twentieth century [31, 32, 81]. Vegetation and flora are diverse [82, 83]. Forests are dominated by *Quercus robur* and *Carpinus betulus* at higher elevations, and *Fraxinus angustifolia* subsp. *pannonica* at lower elevations (see auctors of species in Tables 2 and 3, based on [84]). The health status of *Quercus robur* stands is deteriorating. The herb layer is typical of Central European hardwood forests, but relatively poor in Fagetalia (beech forest) species. Marshes are dominated by *Carex riparia*, *Glyceria maxima*, *G. fluitans*, *Iris pseudacorus* and *Salix cinerea.* Grazed marshes are rich in red-listed Nanocyperion (mud preferring) species such as *Marsilea quadrifolia* and *Ludwigia palustris*.

Organized pig breeding in the vast lowland forest-marsh complex of the Sava river floodplain was integrated into traditional forest management where the beneficial effects of pig rooting on acorn germination were acknowledged by key forestry experts [85]. The pannage fees and rules were designed to be in line with forest management, with several fee rates depending on the development phase of the foraging pigs and the abundance of mast. According to local *svinjar*s, the now extinct traditional breed Sremska Lasa was longer and larger compared to *Mangulica* (Mangalitza), and had a black back, white stomach and shorter hair. It farrowed more piglets than Mangulica. Pigs from the ‘English Pfeifer’ breed were provided to farmers by the state from 1964/1965 to replace the more extensive Sremska Lasa and Mangulica breeds. Mangulica pigs disappeared quickly, but farmers bred Sremska Lasa with Pfeifer and Duroc breeds. Wild boars are present in the area and are hunted. Boars regularly approach pig feeding places and sometimes cross-breed with domestic pigs.

Today, *svinjar*s continue many of the traditional practices but also adapt and innovate them [86]. They keep Yorkshire, Duroc, Landrace and Piétrain breeds, mostly usually their hybrids, which the breeders found to be more resistant and better adapted to year-round outdoor living. The main goal of forest pig keeping is economic (it is cheaper), but the high quality of the meat is also widely valued by *svinjar*s and locals alike (roasted piglets and kulen—a local meaty sausage—is more tasty when prepared from forest pigs). The meat is of similar quality to the famous Iberico ham, but is much less well known. Sows are reported to be healthier and with a remarkably longer period of fertility in the forest (10–12 years) than if kept at home (5–6 years), and they also live longer. “*The forest keeps pigs healthy and cures them.*” The number of pigs and *svinjar*s and the area used by them are decreasing. *Svinjar*s keep their 50–150 pigs (nowadays fewer) in an area measuring ca. 30–100 hectares (< 2000, even < 100 pigs in total in recent years). Since jackals recolonized the area, most *svinjar*s only keep their < 10–30 sows in the forest, while piglets are brought to the village after weaning. Pig numbers kept in the forests were much higher until the 1950–1960s, reaching 30–50 thousands (not rarely hundreds per owner). Additionally cattle and sheep were kept in this area earlier in the twentieth century.

*Svinjar*s live in the nearby villages, keep their own pigs, visit them usually every day for several hours, check their health, and provide extra fodder (mostly corn) as needed (e.g. more in drought and in snow). This regular feeding prevents pigs from wandering larger distances. Pigs are kept in the forest all year round, but shoats born in the forest are often kept at home under semi-intensive conditions on intensive fodder (partly because of the risk of predation by the golden jackal). Even modern breeds can become dangerous, protecting their piglets instinctively, so the owners take care to tame them, in some cases treating them almost as pets. The tameness of the pigs also helped our observational research (see below). During the night, pigs and piglets are kept in massive enclosures built from wood. Pigs are under veterinary control using modern medicines. African swine fever is encroaching on the region. The forest and marsh ‘pastures’ and masting are regulated and contracted from the local forestry office (Internal Code of forest farming, PE Vojvodinašume and PE Srbijašume). The forest is fenced towards the agricultural fields to the north and was previously also fenced to the south (towards non-grazed forests). Habitat management by pigs is part of the current and planned future nature conservation management of the local protected area [31, 32].

### Data collection

We deliberately used an iterative and flexible methodology, combining and alternating indoor and outdoor interviewing, participatory fieldwork, landscape walks and personal visual observation of pig foraging behaviour, in order to reveal as much of the local knowledge related to pigs and plants as possible. As our plan was to conduct exploratory research into knowledge that has hardly been studied before, no quantification was made at this stage. The seasonality of foraging behaviour and foraging on animal species were not focuses of this paper.

After three preparatory visits to the area in 2014–2016 we interviewed 7 highly knowledgeable *svinjar*s about forest and marsh plant species in April 2017. A larger sample size was not possible because the total number of *svinjar*s is itself small (17 persons). Pictures of 234 locally occurring forest, marsh and other plant species (based on the flora list of the area; [83] printed on A4 size sheets were shown to *svinjar*s (plants were shown in a way traditional knowledge holders tend to recognize them: whole plants or focusing on leaves, fruits, rhizomes; see [89]). We asked about the following topics: (1) the local name of the plant; (2) its relationship with pigs (eat, love, avoid, toxic, medicinal etc.), (3) other uses and salient features of the species. The interviewed *svinjar*s were all above 60 years of age, and had lifelong experience with pig keeping. Interviews lasted 2–4 h, and were conducted mostly at the forest huts, in Serbian, constantly translated to English or Hungarian, and transcribed later in full. Prior informed consent was obtained from all *svinjar*s before the first interviews, adhering to the code of ethics of the International Society of Ethnobiology [57] and the GDPR of the European Union [90].

Prior to the interviews, we surveyed the vegetation of the marshes and forests along a grazing intensity gradient (methods and results in [6] and Demeter et al. ined.) to obtain a detailed understanding of the local flora and vegetation, and to help devise specific questions to ask *svinjar*s about foraged (and non-foraged) species and pig foraging behaviour.

We also conducted participatory fieldwork. We joined *svinjar*s during their grazing trips (8 times) and also went on landscape walks with them (12 times). During these occasions we were able to gather data on plants which were not recognized from pictures (e.g. some grasses and small herbs), and discuss pig behaviour on the spot, and observe and discuss how *svinjar*s generated knowledge about plants and pig behaviour.

**Fig. 2:**
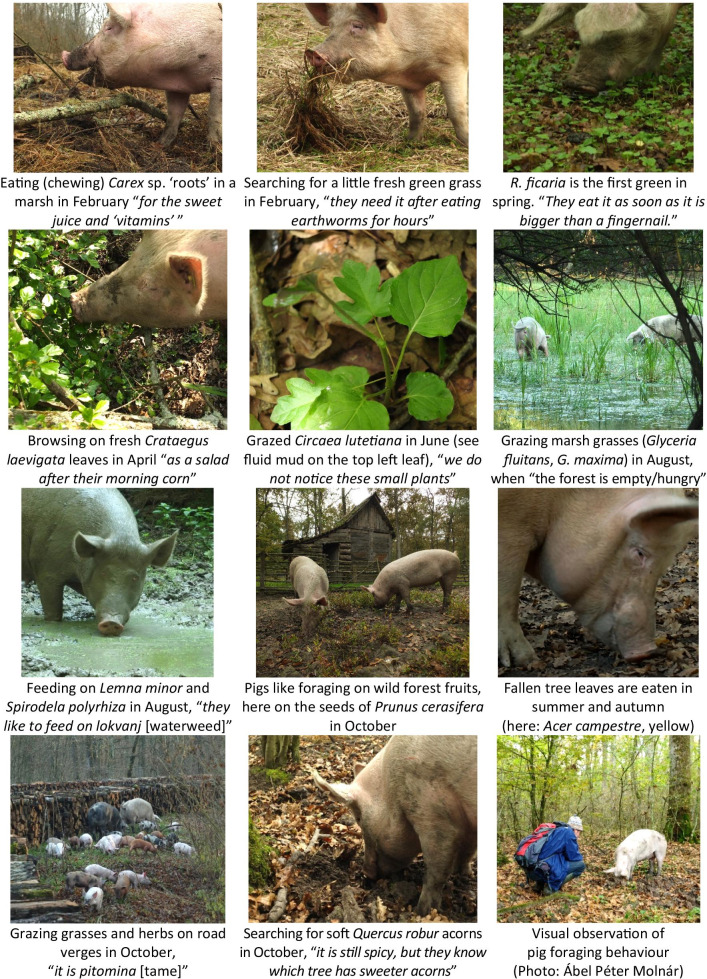
Different types of foraging by free ranging domestic pigs over a year as reported by *svinjar*s and observed by the authors in 2019 in the floodplain forests and marshes of the Bosut and Sava rivers, Serbia (Photos: Zsolt Molnár if not indicated otherwise) (for a video see https://www.youtube.com/watch?v=JJounTmnsXs)

We made visual observations of pig foraging behaviour in February, March, April, June, August, October and December 2019, on altogether 21 days. We followed the herd (3–12 pigs) foraging in forests and marshes, and regularly alternated between the closely observed pig(s), every 5–20 min, depending on their movements. We observed ingestive bite selection and avoidance behaviour from morning till mid/late afternoon (see Fig. 2). Most pigs were calm, and the observed individuals were selected at random. We went as close as possible (0.4–3 m), keeping our impact on the pigs to the minimum while ensuring a good view of the plants near each animal’s mouth. When plants were difficult to identify to the species level, we visited the spot just after the animal moved away. We documented how certain plant species were approached or avoided by pigs and how often and for how long certain species were eaten by them. For the less common species our data are not yet representative for a quantitative analysis, so in Table 2 we indicated the depth and reliability of our data with appropriate wording. To avoid differences among observers, most observations were done by the first author who had a full knowledge of the local flora and was experienced in similar studies [5]. Photos and videos were also made to document foraging. The area regularly visited for data collection covers ca. 750 hectares in total (Fig. 1).

During each visit to observe pig foraging we interviewed *svinjar*s about the foraging behaviour of pigs (1) since our last visit and (2) at the moment, as well as (3) the expected foraging behaviour over the next month. We also shared our experiences collected during visual observations and asked *svinjar*s to comment on those. We used two types of elicitation: we asked direct questions (e.g. what do you think, why were some acorns avoided by this pig?) or formulated statements and waited for *svinjar*s’ comments (e.g. we observed that pigs graze *Ranunculus ficaria* only for some seconds). We also made 5 additional detailed interviews about seasonal changes of foraging behaviour (from January till December). We were also keen to learn how pig keeping and pig foraging behaviour have changed since their childhood (1940s–1960s). Some of the most typical and relevant responses (in translation) are indicated in italicized text between quotation marks.

As the main objective of the research was exploratory, investigating and documenting something which was not considered earlier by ecologists and conservationists, during the whole study we tried to remain as open as possible regarding interview topics and field situations. In some cases our questions might have seemed slightly uncomfortable and unexpected to the *svinjar*s, but they knew that we were keen to understand their knowledge and their culture related to forests, marshes and pigs, so they were very helpful and patient with us. They knew and agreed that the final goal of the research is to publish scientific papers and a book similar to Molnár [89] and Babai et al. [91] on their traditional ecological knowledge and the traditional pig keeping practices and to help the continuation of this still valuable but vanishing land-use practice.

### Data analysis

The 7 main ethnobotanical and 5 season-specific interviews were fully transcribed (520,000 characters). A database was built where data were grouped according to plant species and main topics (foraging behaviour type, season, way of learning about plants, sayings and quotes related to worldview, historical information). All field notes taken during visual observation, participatory fieldwork and landscape walks were also transcribed and inserted into the above database.

We included a plant species in the analysis if at least two independent interview data (i.e. two *svinjar*s) or two independent observations (2 days or two herds) were available. Plant names follow the Euro + Med Plant Base [84]. The following species occur in the study area and/or at least one *svinjar* had some knowledge about them, but they still lack sufficient ethnobotanical or foraging observational data and were therefore excluded from the present analysis (39 species): *Alisma* sp., *Alopecurus pratensis* L., *Artemisia annua* L., *A. vulgaris* L., *Bromus sterilis* L., *Calamagrostis epigeios* (L.) Roth, *Capsella bursa-pastoris* (L.) Medik., *Cardamine impatiens* L., *Fallopia convolvulus* (L.) Á. Löve, *Galium mollugo* L., *G. palustre* L., *Geum urbanum* L., *Gratiola officinalis* L., *Hyosciamus niger* L., *Hypochoeris radicata* L., *Lamium galeobdolon* (L.) Crantz, *L. album* L., *Lapsana communis* L., *Leonurus cardiaca* L., *Myosotis arvensis* (L.) Hill, *Noccaea (Thlaspi) perfoliata* (L.) Al-Shehbaz, *Nostoc* sp., *Ochlopoa (Poa) annua* (L.) H. Scholz, *Pastinaca sativa* L., *Platanthera bifolia* (L.) Rich., *Potentilla anserina* L., *Prunella vulgaris* L., *Ranunculus acris* L., *Rorippa austriaca* (Crantz) Besser, *Sanicula europaea* L., *Sempervivum tectorum* L., *Sinapis arvensis* L., *Stachys sylvatica* L., *Symphyotrichum (Aster) lanceolatum* (Willd.) G. L. Nesom, *Trifolium patens* Schreb., *Valerianella* sp., *Verbena officinalis* L., *Veronica chamaedrys* L. and *V. polita* Fr.

Local frequency of all studied plant species is provided in Table 2 for a general overview of the local flora. Frequency data were based on Perić [83] and the extensive fieldwork of the authors. We regarded ‘roots’ as what *svinjar*s called ‘*koren’* (i.e. roots, bulbs, rhizomes and tubers). We used the following categories of foraging: (1) *eaten/loved*: whole plants, or at least large parts were eaten (if consumption was often observed, it is indicated in Table 2); (2) *nibbled*: only smaller parts were eaten; (3) *avoided*, *not eaten*: untouched on encounter, occasionally smelled (see [5] for more details). Uncertain but probably relevant data were put into parentheses in order not to lose any already identified information in this exploratory study.

Categories of knowledge depth for the 192 analysed plant species: (1) *well known*: all or most (5–7) *svinjar*s knew its local folk name(s) and could describe the species in detail; (2) *moderately known*: only 3–4 *svinjar*s knew the species, and/or knowledge was less detailed; (3) *little known*: only 1 or 2 *svinjar*s knew the species or if more, knowledge was not detailed (usually rare or small or ‘insignificant’ species); (4) *not known*: none of the *svinjar*s knew the species (these species were only included in Table 2 if visual foraging observation data by the authors was available). The level of local knowledge of each species was compared to the regional level knowledge of the species (from well known to not known in other areas of the Carpathian Basin) using Molnár [89], Dénes et al. [92], Babai et al. [91], and Molnár [93].

## Results

### *Svinjar*s’ knowledge of plants and observations on pig foraging behaviour

We collected ethnobotanical and/or visual observation data for 237 species, of which 192 could be analysed (i.e. had two independent data). *Svinjar*s knew 181 of the 192 species. 102 plant species were well known by them (Table 1), 27 species were moderately known, 52 little known, and 11 species that occurred in the forests and marshes were probably not known by them (Tables 2 and 3). There were 10 species that were well or moderately known but not named by *svinjar*s. *Svinjar*s regretted that they could not remember any name.

**Table 1:** Overview of the number of plant taxa: *svinjar*s’ plant knowledge and authors’ observations, species that were known by *svinjar*s as eaten or avoided by pigs and/or observed as eaten or avoided by pigs

Categories of knowledge depth	Number of plant taxa (from the analysed 192)		
	Trees and shrubs	Grasses, sedges and rushes	Others (herbs etc.)
Well known	28	12	62
Moderately known	3	3	21
Little known	6	5	41
Not known	0	1	10
Total	37	21	134

Pig foraging behaviour	*Svinjar*s’ knowledge	Authors’ observations	Overlaps
Regularly eaten, loved	61	28	21
Nibbled, rarely eaten	37	21	10
Not eaten	56	17	8
No knowledge by *svinjar*s / No observation by the authors	38	126	10
Total	192	192	49

**Table 2:** *Svinjar*s’ knowledge of wild plant species and visual observations by the authors of pig foraging of the studied plant species in the Sava-Bosut floodplain, Serbia

Latin name, (local frequency), depth of local knowledge, folk names, (depth of knowledge regionally—Carpathian Basin)	Pigs eat/don’t eat (*svinjar*s’ reports)	Pigs eat/don’t eat (authors’ observations)	Other uses and salient features (e.g. impact on pigs) reported by *svinjar*s
**Trees**			
*Acer campestre* L. (3), well known, *klen* (well known)	Leaves are eaten eagerly in spring, liked more than *Carpinus* leaves, eaten less in summer but longer than other tree leaves, also fallen leaves in autumn, fruits are eaten in need	Leaves were eaten, also fallen leaves	Leaves have a sweet taste
*Acer tataricum* L. (2), well known, *žesta* (fierce, spicy) (moderately known)	Neither leaves nor fruits are eaten	(It was nibbled)	Common in forest and marsh edges, short-lived, resprouts and can have several trunks, has a different fruit than *Acer campestre*
*Carpinus betulus* L. (4), well known, *grab* (well known)	Leaves are liked and eaten, but much less in summer, also buds and young twigs, seeds and germinating and soaked seeds are eaten eagerly for whole days from late autumn till spring if there is no acorn	Leaves, young twigs and seeds were eaten	Leaves are bitter, seeds (size and form like apple seeds or sunflower seeds) are healthy for piglets (stomach and lung), seeds are not nutritional at all and too small to provide enough forage for larger pigs (“Happiness for the mouth but sorrow for the ass.”), *Carpinus* spreads because there is no cattle grazing in the forest, wood is good for firewood and tool handles
*Prunus avium* (L.) L. (1), well known, *trešnja* (cherry) (well known)	Fruits are eaten (domestic cherry)	N.o	Not in the forest, the domestic type is good for jam
*Fagus sylvatica* L. (0), well known, *bukva* (well known)	In the past, the black pigs were driven for masting to neighbouring mountains	N.o	It does not grow here, people went to Bosnia, Montenegro, Fruška Gora mountain (especially when the corn harvest was poor), mast was also eaten by people
*Fraxinus angustifolia* Vahl (3), well known, *jasen* (well known)	Leaves on trees are not eaten, fallen twigs and leaves are eaten, fruits are not eaten	Leaves on trees were not eaten (sometimes nibbled), fallen leaves were eaten	Dry *Fraxinus* is the best firewood, pigs eat the leaves if acorns are spicy, there is more *Fraxinus* since the red deer population decreased
*Fraxinus pennsylvanica* Marshall (2), little known, *Amerikanac/divlji jasen* (American/wild ash) (little known)	N.d	N.o	Grows along waters
*Juglans nigra* L. (1), moderately known, *divlji orah* (wild walnut) (moderately known)	Nuts are eaten	N.o	A planted tree [rarely]
*Juglans regia* L. (1), well known, *nežni orah* (tender walnut) (well known)	Nuts are eaten, leaves are not eaten	N.o	Rare, squirrels eat nuts, goats eat the leaves
*Malus sylvestris* (L.) Mill. (2), well known, *divlja jabuka* (wild apple) (well known)	Fruits are eaten but less and starting later than *Pyrus* fruits, more till acorns fall or when there is no acorn (the forest is empty), leaves are nibbled	Leaves were eaten once, fruits: n.o	Used for vinegar
*Morus alba* L. (2), well known, *dud*, fruits: *dudinje* (well known)	Fruits are eaten, leaves are nibbled, fallen leaves are eaten	N.o	Planted around forest huts, chicken and geese like its fruits, there is black and white, good for *rakija* (brandy)
*Populus alba* L.*, P. nigra* L.*, P.* × *canadensis* Moench*, P. tremula* L. (2), well known, *bela topola* (white poplar), *crna topola* (black poplar), *Kanadska topola* (Canadian or hybrid poplar), *jašika/jošika* (aspen) (well known)	It is not eaten, rarely the young leaves are eaten	N.o	Wood is good for boards, and for making a bread trough
*Prunus cerasifera* Ehrh. (2), well known, *džanarika, zerželija, divlja šljiva* (wild plum) (well known)	Fruits, later seeds and less the leaves are eaten	Seeds were eaten	Used for *rakija* (brandy), jam, eaten fresh by people
*Pyrus* spp. (wild and ancient semi-wild) *(*2), well known, *divlja kruška* (wild pear) (well known)	Fruits are eaten eagerly from late August till acorns fall, and eaten up entirely if there is no acorn (till spring), but not if ‘not needed’, preferred to wild apple, pigs wait 2–3 days for after-ripening of fallen fruits, some trees are only eaten later, leaves are eaten (by male pigs)	N.o. (the fruit of a “bad” tree was not eaten)	Grows in open forests and around marshes, trees with bad tasting fruits are avoided, eaten by people, used for *rakija* (brandy), *turšija* (pickles) and jam, also vinegar, healthy, there are ancient (grafted), not real wild pear varieties, these are ancient types of domesticated pears, not wild, sweet as honey
*Quercus cerris* L. (1), well known, *cer* (well known)	Acorns are eaten, but only in spring after they are wettened and softened and not if *Q. robur* acorn is available, leaves are rarely eaten	N.o	Rare here, very spicy, worth half compared to *Q. robur* acorns, may cause miscarriage in pigs, pigs were driven to its acorns if *Q. robur* acorn was not available, wild boars also prefer *Q. robur* acorns, acorns burn well in the stove or furnace, wood is less valuable than of *Q. robur*
*Quercus robur* L. (5), well known, *hrast, lužnjak,* acorn: *žir* (well known)	Acorns are very much liked, the primary food of pigs, unripe, recently ripened and dry acorns are eaten less, are very spicy, liked if soaked by rains by November, pigs know where to find the trees fruiting the sweetest acorns, pigs search for acorns in wood mice’ stores under stumps (*nado* = the store), acorns fallen into marshes are kept fresh and eaten months later, cotyledons of germinated and thus very sweet acorns are uprooted, leaves are eaten, but only when young, and only the freshest leaves from twigs fallen in summer, otherwise too bitter, home-kept pigs eat even dry leaves (of *badnjak*)	Acorns were eaten if available, germinating acorns were preferred, leaves were not eaten, only young leaves	There are early and late trees, acorn production is forecasted by *svinjar*s from late spring onwards, frost kills flowers, till the day of the Apostles Peter and Paul (12th July in the Orthodox calendar) acorns only grow on Saturdays, later every day, Saint Elijah (hot days in July) kills acorns, acorn is as good for pigs as *kulen* (very meaty sausage) is for us, but pigs need enough water, fatten less on spicy acorns, acorns get sweeter and softer after rains, acorns used to be shaken off from early ripening trees in August, collected in October, kept in stacks and given to home-kept or to forest pigs, trees are less productive and healthy nowadays, acorn is collected for forestry. The bacon fat is getting yellowish and firm from the acorn pannage
*Robinia pseudoacacia* L. (2), well known, *bagrem* (well known)	Leaves even if fallen are not eaten, nor flowers or pods	N.o	Not a common tree here, medicinal tea, we ate the flowers, honey plant, sheep eat leaves and pods, has good timber
*Salix euxina* I. V. Belyaeva [“*fragilis*”] (3), well known, *vrba, bela* (white) *vrba* (well known)	Young and fallen leaves are eaten	N.o	Twigs break easily, used for whistles [in spring]
*Tilia tomentosa* Moench, *T. platyphyllos* Scop. (1 + 1), moderately known, *lipa* (well known)	It is not eaten	N.o	In the village, honey plant, the wild variant blooms later and has smaller leaves
*Ulmus laevis* Pallas (2), well known, *vez* (ties, bond) (well known)	Leaves are not eaten, seeds are eaten a bit	N.o	Not very useful, not planted
*Ulmus minor* Mill. (2), well known, *brest* (well known)	Young leaves and fallen seeds are eaten but not eagerly	Leaves were eaten once	Not very useful, a good mushroom grows on it (*Pleurotus ostreatus*), farmers used to cut and leave a log for the mushroom to develop
**Shrubs**			
*Amorpha fruticosa* L. (3), well known, *divlji bagremac* (wild *Robinia*), *fašina* (bundle of loppings) (well known)	Leaves are not eaten, nibbled when young	N.o	“Poisons” (i.e. overgrows) marshes, pigs avoid such places, cattle, sheep and red deer eat, bees like it, used for baskets
*Cornus mas* L. (1), well known, *dren* (well known)	Fruits are eaten	N.o	There isn’t much here, medicinal, blooms early but fruits late, fruits used for jam and put into *rakija* (brandy), making strengthening medicine, wood good as herder sticks and tomato stakes
*Cornus sanguinea* L. (3), well known, *sibovina, svibovina, svib* (related to May) (well known)	It is not eaten, leaves are nibbled when young, fruits eaten in need	N.o	Wood is flexible, good for fences, long-lasting in wattle-and-daub walls, baskets, brooms, but not as herder sticks because it causes goitre to pigs, white-flowers, fruits paint your skin blue but cause itching
*Corylus avellana* L. (2), well known, *lešnik* (well known)	Fallen fruits are eaten, leaves are not eaten	N.o	People also eat fruits
*Crataegus monogyna* Jacq., *C. laevigata* (Poir.) DC. (4 + 4), well known, *glog*, rarely two types were distinguished (the two species), fruit: *gloginje* (well known)	Leaves are eaten when fresh and spikes are soft, a good ‘salad’ after morning corn for pigs, fallen fruits are eaten especially in snowy winter	Leaves were regularly and patiently (1–3 min) eaten in spring and summer, pulled down from twigs, n.o. for fruits	Likes open forests and open places, thorns disturb pigs, flowers are medicinal, *C. laevigata*: has looser crown structure, blooms earlier, and grows in forest interiors, it was thrown onto water and fish were caught in the shade, good for herder sticks, a plant of the devil
*Euonymus europaeus* L. (2), little known, uncertain names only (moderately known)	(It is not eaten)	N.o	Rare, grows one by one
*Prunus spinosa* L. (2), well known, *trnjina* (thorny) (well known)	Leaves are not eaten, fallen fruits are eaten in autumn and winter	Leaves were rarely nibbled	Good for *rakija* (brandy), berries are also eaten by people after the first frost, used for refreshing tea
*Rosa canina* L. (2), well known, *divlja ruža* (wild rose), fruit: *šipurak, šipkovina* (well known)	Leaves are not eaten, too spiky, fruits are rarely eaten, in winter when the forest is ‘empty’	N.o	Along roads, canals, in forest edges, not in the forest, prickles disturb pigs, used for jam, tea, put into *rakija* (brandy), healthy, petals soaked in water
*Rubus caesius* L., *R. hirtus* aggr. (3 + 2), well known, *jagode* (strawberry), *pepeljuga* (Cinderella), *kupina, ostruga* (scrape off) (well known)	Leaves are eaten especially in winter, and rarely the fruits	Leaves were eaten, also in summer	Leaves stay green in winter, grows along roads and forest edges, spreads as livestock numbers decrease, the wild type is more tasty, good for jam, roe deer love it
*Ruscus aculeatus* L. (1), moderately known, *divlji šimšir* (wild boxwood) (moderately known)	It is not eaten	N.o	Rare here, green in winter, red deer doesn’t eat it either, ornamental, collected, used to keep away mice from drying meat hung on rods and to block mouse holes
*Salix cinerea* L. (3), well known, *ivovina, ivovina vrba*, a kind of *vrba* (willow) (well known)	It is not eaten, neither the roots	N.o	In marshes, medicinal, deer eat it and clean their antlers on its twigs (thus may kill bushes), if catkins are taken into the house, hens stop laying eggs, used for baskets and whistles
*Sambucus nigra* L. (2), well known, *zova* (well known)	It is not eaten, neither leaves nor fruits (seeds/fruits are eaten rarely)	N.o	For pigs the only use is that they like its shade…, medicinal, used for syrup, *rakija* (brandy) and jam, red deer, cattle and sheep eat it, a mushroom good for “elderly men’s problem” grows on it
*Viburnum opulus* L. (2), little known, *kereće grožđe* (dog grape) (little known)	It is not eaten	N.o	Has red berries
*Vitis* sp. (non-native) (1), moderately known, *divlja loza* (wild vine) (well known)	It is not eaten, neither leaves nor fruits	N.o	Grows around marshes
**Forest herbs**			
*Ajuga reptans* L. (4), well known, *ranjenika/ranjena/ ranjenik trava* (grass of the wounded) (n.d.)	It is not eaten, nibbled when young	It was not eaten, but nibbled in winter	In forests, medicinal for wounds, calluses, pus, pimples and stomach, it stays green for the winter so that(!) people in need can find it under the snow
*Allium ursinum* L. (0), well known, *sremuš* (belongs to the Srem region) (well known)	N.d	N.o	Doesn’t grow here, only in the mountains, edible and healthy, people collect the leaves
*Athyrium filix-*femina (L.) Roth, *Dryopteris filix-mas* (L.) Schott (2 + 2), well known, *paprat* (well known)	It is not eaten, not even the roots	It was not eaten	In wet parts of the forest, in wells, green for the whole year, pretty, collected for bouquets, I have watched it for long – it has no flower!
*Carpesium abrotanoides* L., *C. cernuum* L. (2 + 1), little known (n.d.)	N.d	It was not eaten (but smelled)	
*Circaea lutetiana* L. (3), little known (n.d.)	N.d	It was regularly eaten, for seconds, max. 2 min	Too small for us to notice…
*Clematis vitalba* L. (1), moderately known, *divlja loza* (wild vine), mistaken for *Humulus* (n.d.)	N.d	N.o	We played Tarzan…
*Convallaria majalis* L. (1), well known, *đurđevak* (George flower, blooming around Orthodox St. George’s day), *zvončići* (small bells) (well known)	It is not eaten	N.o	Grows in patches, collected by people, fragrant, medicinal
*Ficaria verna* Huds. (4), well known, *pšenac* (tubers are similar to wheat grains: pšenica), rarely: *kopitnjak* (horse shoe) (well known)	It is eaten as soon as it is bigger than a fingernail, less eaten if in bloom (bitter), flowers are not eaten, available till 10th May, bulbs are eaten in autumn but not eagerly	Leaves were eaten regularly and often patiently for 0.5–3 min	Grows in early spring, this is the first fresh green in the forest, has a bitter taste, has small bulbs like a wheat grain but they only make the pigs’ hair grow (not the meat)
*Galanthus nivalis* L. (1), well known, *visibaba* (hanging grandma) (well known)	It is not eaten	N.o	Rare here, blooms at around snowmelt, makes you happy, disappears by summer, flowers are picked, otherwise not useful
*Hedera helix* L. (3), well known, *bršljen, bršljan* (browsed), a kind of *puzavica* (creeper) (well known)	It is eaten especially in winter, fruits are not eaten	It was not eaten	Climbs on trees, “poisonous” for trees, blooms in late autumn, you can easily stumble on it, red and roe deer, cattle, goat and sheep also eat it, bees visit flowers
*Leucojum aestivum* L. (2), moderately known, *barska visibaba* (marsh *Galanthus*), *letnja visibaba* (summer *Galanthus*), *zvončić* (bells) (moderately known)	Neither leaves nor flowers are eaten, only the roots, and pigs like rooting around it	It was not eaten	In flooded places, has larger flowers than *Galanthus*, slightly fragrant (picking by people was not mentioned)
*Moehringia trinervia* (L.) Clairv. (3), little known, *mišovina, mišovkinja* (part of the *Stellaria media* folk taxon) (n.d.)	It is eaten	It was eaten once	Grows both in forests and fields (cf. *Moehringia* and *Stellaria*), has small white flowers
*Neottia nidus-avis* (L.) Rich. (2), little known, name not remembered (n.d.)	It is not eaten	N.o	It looks as if it has dried out (always brown), has a thick and hollow root
*Potentilla indica* (Jacks.) Th. Wolf (1), little known, rarely: divlja jagoda (wild strawberry), (n.d.)	It is not eaten	N.o	A newcomer, game eats it
*Primula acaulis* (L.) L. (3), well known, *jagorčevina* (well known)	Leaves are eaten when young / are not eaten	N.o	Grows in early spring, medicinal, flowers are used in *rakija* (brandy)
*Rumex sanguineus* L. (3), moderately known, kind of *zelje*, *štavalj* (material for tanning, ancient meaning is sour, tart)*, masnih* (greasy on touch) (little known)	It is eaten	It was eaten once	Sour
*Scilla bifolia* L. (3), well known, *procepak, pricepak, precepak* (split), *divlji zumbul* (“wild hyacinth”) (moderately-well known)	It is never eaten	N.o	Blooms in early spring in the forest, after *Galanthus* and before *Viola*
*Scrophularia nodosa* L. (2), little known (little known)	It is not eaten	It was not eaten	
*Veronica montana* L. (3), little known (n.d.)	(It is not eaten)	It was eaten once, was also avoided	
*Viola alba* Besser (2), well known, *bela* (white) *ljubičica* (from ‘ljubica’ = loved /dear woman) (well known)	(It is eaten) (It is not eaten)	N.o	
*Viola reichenbachiana* Boreau (3), well known, *plava* (blue) *ljubičica* (well known)	(It is not eaten) (It is eaten)	It was eaten (in August regularly), sometimes avoided	Pigs wish for this green
**Wetland plants, waterweeds**			
*Butomus umbellatus* L. (2), moderately known, *koštan* (bony) (moderately known)	Roots are eaten	N.o	It has white roots like onions or small potatoes
*Callitriche* sp. (2), little known, some relate it to *Lemna* spp. (n.d.)	(It is eaten) (unsure, dubious)	N.o	Stays green on mud after water dries up
*Ceratophyllum* spp., *Myriophyllum* spp. (2), well known, *drezga*, a kind of *lokvanj* (moderately known)	It is eaten in small amounts or in food shortage, pigs swim to get it	N.o	A bit spiky, a lot in the Bosut river and on its shores, also in deeper marshes, spreads nowadays as the water is polluted
*Iris pseudacorus* L. (3), well known, *perunika* (from Perunika, the wife of Perun, the supreme Old Slavic deity) (well known)	Roots are eaten in summer, late autumn and winter when there is no water in the marshes, leaves are not eaten, flowers are not eaten	Leaves: nibbled regularly, roots: n.o	Roots are grown together like potatoes (in a chain), has reddish seeds
*Lemna* spp., *Spirodela polyrhiza* (L.) Schleid. (2), well known, *sočica* (lentil), *sočivica* (lentil) (well known)	It is eaten from the water surface, pigs also swim for it in Bosut	It was eaten eagerly, for several minutes	In marshes, may cover the whole water surface, bad for the fish (less oxygen), survives on the mud, deer and cattle eat it
*Marsilea quadrifolia* L. (1), little known, *barska detelina* (marsh clover) (n.d.)	(It is eaten but only a bit) (It is not eaten)	It was nibbled once	Like a four-leaf clover, not common
*Nuphar lutea* (L.) Sm. (1), moderately known, *žuti* (yellow) *lokvanj* (puddle weed) (moderately known)	Roots are eaten (leaves and flowers are eaten a bit)	N.o	In marshes and canals, disappeared from drying marshes
*Nymphaea alba* L. (1), well known, *beli* (white) *lokvanj* (well known)	Roots are eaten, (leaves and flowers are eaten a bit)	N.o	Herbicides killed them off
*Oenanthe aquatica* (L.) Poir. (1), little known, a kind of *kukuta* (*Conium*) (little known)	It is eaten (It is not eaten)	It was regularly eaten (also in winter)	
*Potamogeton* spp. (2), little known, *drzega*, a kind of *lokvanj* (little known)	It is not eaten	N.o	
*Rorippa amphibia* (L.) Besser (2), little known (little known)	N.d	It is eaten (young and older leaves as well), even from below the water	
*Trapa natans* L. (1), well known, *rašak, orašak* (little walnut), fruit: *krava* (cow), *šišarka* (pine cone) (well known)	Green and ripe fruits are eaten with pleasure, pigs swim for it, even eat fruits from last year, dried black fruits are not eaten, roots are eaten, leaves are less eaten	N.o	In deep water, extinct here, pigs fatten on the fruits, inside: like a hazelnut, edible for humans, painful for bare feet
**Grasses and sedges**			
*Agrostis stolonifera* L. (3), well known, *mekuša* (softy) (well known)	It is often eaten and all year round	It was often and patiently (for 5–10 min) eaten all year round, a basic forage in summer and autumn	Grows in and around marshes, regrows and green also in autumn
*Brachypodium sylvaticum* (Huds.) P. Beauv. (4), little known (n.d.)	It is nibbled	It was nibbled	Soft
*Carex elata* All. (1), well known, *šaš* (well known)	It is eaten less often than other marsh sedges, only if in need, roots are not eaten	N.o	Grows like *Juncus* (= tussocky), deer eat it
*Carex riparia* Curtis, *C. vesicaria* L. (3), well known, *šaš*, there are different types (well known)	Roots are eaten especially in winter when they are ‘ripe’, pigs suck out ‘vitamins’ and ‘juice’, green and young leaves are eaten in winter, especially in deep snow, they eat to eat something, it goes through them (undigested)	Roots and leaves were eaten, especially in winter, 10–30 cm long roots were uprooted and chewed for 2–3 min	Grows in marshes, people used to make ropes for wheat harvest, used as hut roof, leaves cut your finger, fruits are rare, red deer eat it
*Carex sylvatica* Huds., *C. remota* L., *C. divulsa* Stokes (2 + 2 + 3), moderately known, *sitni* (small) *šaš*, *trava* (grass), *štitasit* (little known)	It is eaten if other greens are dry in autumn and spring, and especially in snow, it is not eaten in summer	It was eaten but not intensively, sometimes a whole tussock, only nibbled in summer	Grows in forest, this is a kind of forest grass
*Cynodon dactylon* (L.) Pers. (1), well known, *zubača* (toothy), there are two kinds if it (well known)	Roots are eaten, especially in winter, also leaves, young leaves are preferred	N.o	On arable land, on road verges and pastures, less in forests, resistant to herbicides, can revive itself after 7 years spent in the attic
*Deschampsia cespitosa* (L.) P. Beauv. (1), little known (little-moderately known)	It is eaten, also in winter	N.o	
*Echinochloa crus-galli* (L.) P. Beauv. (2), moderately known, *sirak, divlji sirak* (wild sorghum) kind of *muhar* (well known)	It is eaten when fresh	N.o	Grows in corn fields, but also in forests in open wet places
*Eleocharis palustris* (L.) R. Br. (2), little known, *sita* (tie), a kind of *sita* (moderately known)	Roots are eaten in winter	Was only nibbled, roots: n.o	
*Elytrigia repens* (L.) Nevski (1), well known, *zubača* (toothy), *pirevina*, there are two kinds of it (well known)	Roots are eaten especially in winter, also the leaves, collected from fields and given to forest pigs	N.o	Weed in arable land, less in the forest, survives even if kept in the chimney for 9 years, resistant to herbicides
*Glyceria fluitans* (L.) R. Br. (2), little known (related to *Agrostis*) (little known)	It is eaten regularly	It was often grazed all year round and patiently for minutes, in dry and also in water-logged marshes	Grows in marshes
*Glyceria maxima* (Hartm.) Holmbg. (3), moderately known, sometimes part of the *Carex* taxon (little known)	Roots are eaten, also leaves	Leaves were regularly eaten	By grazing on it, pigs compensate the effects of acorn (acorn is a heavy food), red deer eat it
*Juncus effusus* L. (2), well known, *sita*, there are different kinds of *sita* (tall and small) (moderately known)	Roots are eaten in winter, whole plants may be uprooted, leaves are rarely eaten, in snowy winter and when fresh, eaten by pigs in need, i.e. who are not fed properly with corn	It was avoided or nibbled, roots: n.o	Not useful, it was used in the past to seal gaps between boat planks
*Leersia oryzoides* (L.) Sw. (1), not known (n.d.)	N.d	It was eaten several times	
*Lolium perenne* L. (2), well known, *muhar, vlasulja* (with long hairs), uncertain names (well known)	It is eaten when young, less when in flower	N.o	On fields and meadows, along roads, in 4–5-years-old clover fields, tame/sweet grass, cattle and sheep like it, also dogs
*Phragmites australis* (Cav.) Steud. (2), well known, *trska* (well known)	Maybe would be eaten (not occurring in the local study area)	N.o	Needs water, there is none here, there were more in the past, farrowing sows liked to hide in it (protection from jackals and foxes), used for roofs, water is drinkable in reed beds
*Poa pratensis* L., *P. trivialis* L. (2 + 2), little known (moderately known)	It is eaten when fresh	It was eaten several times	
*Schoenoplectus lacustris* (L.) Palla (2), well known,*’prava’* (real) *siita* (well known)	Roots are eaten in summer, even under water, in soft mud, but also in winter, especially if there are no acorns, leaves are not eaten	It was regularly avoided	It lives in water, good for baskets, bad as roofing, has small brown flowers on the top
*Setaria* spp. (2), well known, *mu’ar, krpiguz* (ass patching), *bodljikavo prase* (spiny piglet, meaning hedgehog) (well known)	It is eaten when young, we grazed it in summer in the past	N.o	Grows in the fields, not in the forest, sticks to clothes (only the seeds)
*Sorghum halepense* (L.) Pers. (1), well known (well known)	It is not eaten	N.o	Total herbicide is used against it
*Typha angustifolia* L., *T. latifolia* L. (1 + 1), well known, *rogoz* (horny), spike: *keka, palčika* (stick), *palačka* (well known)	Roots are eaten / roots are not eaten (contradicting reports)	N.o	Grows in water, in canals, non-flowering individuals were used for ropes (strong, wide leaves and doesn’t cut like sedge), for sealing barrels, ornamental, children’s toy
**Other generalist dicotyledons**			
*Allium scorodoprasum* L. (1), moderately known, *luk* (well known)	N.d	N.o	Smells like onion or garlic, edible
*Althaea officinalis* L. (2), well known, *beli* (white) *slez* (well known)	It is not eaten	Small bits were nibbled	In wet places, medicinal, collected for sale
*Ambrosia artemisiifolia* L. (2), well known, *ambrozija* (well known)	It is not eaten	N.o	Causes allergy, has a bad smell, a bad weed, doesn’t grow in the shade, sheep and goats eat it
*Anthriscus cerefolium* (L.) Hoffm. (2), little known, a sort of *kukuta* (*Conium*) or *peršun* (*Petroselinum*) (little known)	It is not eaten	N.o	
*Arctium lappa* L. (2), well known, *repuh, veliki čičak* (big bur with hooks, big sticking bur) (well known)	Leaves are eaten but only in very early spring, roots are eaten in winter	Plants were avoided	In sparse forests, children use it as an umbrella and throw burs into girls’ hair, medicinal, deer eat it
*Asclepias syriaca* L. (2), well known, *divlji pamuk* (wild cotton) (well known)	It is not eaten	N.o	Along roads and dykes, honey plant, stalks are bad in hay
*Astragalus glycyphyllos* L. (2), little known (little known)	It is not eaten	It was not eaten	Not even goats eat it
*Ballota nigra* L. (3), moderately known, relative of *mrtva žara* (dead ember), *mrtva kopriva* (dead nettle) (= *Lamium* sp.) (moderately known)	It is nibbled a bit in need when young	N.o	Smaller than the nettle, honey plant, it is used for catching wild (and fleeing) bees by its smell
*Bidens tripartitus* L., *B. frondosus* L. (3), well known, *viljuščica* (small fork), *mali čičak* (small bur with hooks) (well known)	It is eaten when young	Whole plants were eaten when fresh, incl. flowers and young fruits	Grows in abandoned wet places, easily sticks to clothes
*Caltha palustris* L. (1), well known, type of *ljutić* (spicy) (well known)	It is not eaten, pigs walk across it	It was not eaten	Grows in marshes, has star-like fruits
*Calystegia sepium* (L.) R. Br. (1), well known, *poponac* (climber), forms a folk taxon with but distinguished morphologically from *Convolvulus* (well known)	It is eaten	N.o	Grows in weedy arable fields, in marshes, rare in forests
*Cardamine pratensis* L. (2), moderately known, name not remembered (n.d.)	It is not eaten, rarely nibbled when young	It was both nibbled and avoided several times	It is rare in the forest
*Chaiturus (Leonurus) marrubiastrum* Ehrh. ex Rchb. (1), little known (n.d.)	(Rarely nibbled a bit)	It was not eaten	
*Chenopodium album* L., *Lipandra polysperma* (L.) S. Fuentes & al. (2 + 2), well known, *zelje* (greeny, a common name for juicy leafy weeds) (well known)	It is eaten when fresh, but also the seeds, it was often collected for green fodder in the past for home and forest pigs, causes diarrhoea to home-kept pigs	N.o	Grows in arable fields, rare in forests, mostly in former fodder places, all livestock like it, some people eat it cooked
*Cichorium intybus* L. (2), well known, name not remembered, a *korov* (weed) (well known)	It is not eaten	N.o	In fields, along roads, not in forests, medicinal, honey plant
*Cirsium arvense* (L.) Scop. (2), well known, *‘prava’* (the real) *boca* (spiny), *čičak* (sticking bur), *palamida* (well known)	It is eaten only when young, flowers are not eaten, maybe roots are also eaten	N.o	Noxious weed, rare in forests, honey plant
*Cirsium vulgare* (Savi) Ten., *Carduus acanthoides* L. (1 + 1), moderately known, magareća/magarca *trava* (donkey grass), *čičak* (sticking bur), *boca* (pricker) (well known)	It is eaten only when young	N.o	
*Convolvulus arvensis* L. (2), well known, *poponac* (climber), forms a taxon with but distinguished from *Calystegia*, *slatkovina* (sweet), *slatkiš* (candy) (well known)	It is eaten	N.o	In arable fields, rare in forests
*Dipsacus fullonum* L. (1), well known, *češlja* (comb) (well known)	It is not eaten	N.o	In abandoned places, not in the forest, you can drink from it, nothing eats it, honey plant
*Erigeron annuus* (L.) Desf. (2), little known (n.d.)	(It is not eaten)	It was eaten regularly	
*Euphorbia* spp. (2), well known, *mlečika* (milkweed), several types, species are not well distinguished, kind of *paprat* (well known)	It is not eaten	(It was not eaten)	It leaks milk when broken
*Fragaria vesca* L. (2), well known, *divlja jagoda* (wild strawberry) (well known)	Fruits are eaten (but is not an important forage), leaves are not eaten	N.o	Grows in drier sunnier places, fruits are eaten by people, smells good, wild boar avoids its fruits, used for *slatko* (fruit put into sweet syrup)
*Galium aparine* L. (2), well known, *krpiguz* (ass patching), *prilepača* (sticks to), *čičak* (sticking bur) (well known)	It is not eaten	N.o	Sticks to you
*Genista tinctoria* L. (1), little known (n.d.)	It is not eaten	N.o	Along forest roads, deer eat it, not a tree but not a herb either
*Geranium* spp. (annual spp.) (2), little known (moderately known)	Unsure	It was not eaten	
*Glechoma hederacea* L. (4), little known, it has no local name (n.d.)	It is not eaten, only rarely eaten	Leaves were regularly eaten but only for < 1–2 min, sometimes also the roots	Too small for us to see…
*Humulus lupulus* L. (1), well known, *hmelj* (well known)	It is not eaten	N.o	Climbing plant
*Hypericum hirsutum* L., *H. tetrapterum* Fr. (1 + 2), little known (well known)	(It is eaten)	It was eaten	
*Lamium purpureum* L. (2), well known, *mrtva kopriva* (dead nettle), *mrtva žara* (dead ember) (well known)	It is eaten when young, pigs are not keen on it, don’t adore it	N.o	In open places, not in forest, honey plant
*Lathyrus tuberosus* L., *L. pratensis* L. (1 + 1), well known, *divlji grašak* (wild pea) (well known)	It is eaten	N.o	In arable fields and clear cuts, climbing plant, cattle also eat it (bulbs were not eaten by children)
*Linaria vulgaris* Mill. (2), little known, name not remembered (moderately known)	(It is not eaten)	N.o	In grasslands, we played with it (open-close)
*Lycopus europaeus* L., *L. exaltatus* L. Fil. (2 + 2), not known (little known)	N.d	It was not eaten, was avoided, nibbled once	
*Lysimachia nummularia* L. (3), little known (n.d.)	(Usually it is not eaten, leaves and roots are eaten rarely)	It was eaten, also avoided, roots were also eaten	In wet places
*Lythrum salicaria* L. (2), moderately known (little known)	It is not eaten	N.o	In marshes and canals
*Melilotus albus* Medik. (2), moderately known, *divlja detelina* (wild clover), *smrdljan* (smelly) (well known)	It is not eaten	N.o	
*Mentha aquatica* L., *M. longifolia* (L.) L. (3 + 1), well known, *konjski bosiljak* (horse basil), the two species are partly distinguished (well known)	It is not eaten (rarely nibbled)	It was not eaten, nibbled once	In margins of marshes, honey plant, has a good and strong smell, good for tea, useful against mosquitos
*Myosotis scorpioides* L. (2), little known (moderately known)	It is not eaten	N.o	
*Persicaria dubia (P. mite)* (Stein) Fourr. (4), well known, *divlja paprika* (wild pepper), *paprat* (meaning a useless plant) (moderately known)	It is eaten if no better is available, e.g. in drought, or while fresh, on sunny spots	It was eaten regularly, but more often avoided, after smelling	In wetter places, spicy, plants like *P. dubia* age later as they live in wet places
*Physalis alkekengi* L. (2), well known, *gujina* or *divlja jabučica/jabuka* (snake or wild apple) (well known)	It is not eaten	It was not eaten	We like and eat it, very healthy, if red, full of vitamin C, one berry equals one-two lemons, sour-bitter, it is sold in shops
*Phytolacca americana* L. (1), well known, name not remembered (n.d.)	It is not eaten	N.o	Grows along roads, has a hollow red stem
*Plantago major* L. (3), well known, *bokvica* (well known)	It is eaten (while fresh) (It is not eaten)	N.o	Medicinal for wounds
*Polygonum aviculare* L. (3), well known, *troskot, troskut, troskoč, troskovača* (well known)	It is loved by pigs, cannot grow fully, it is eaten up, it is eaten less when it is older	It was eaten eagerly	On (dirt) roads, in yards, creeping on the ground
*Potentilla reptans* L. (2), well known, like *jagodnjak* (strawberry), *riblja trava* (fish grass) (moderately known)	It is eaten eagerly	(It was not eaten)	In flooded grasslands, similar to *Fragaria* but with no fruits
*Ranunculus repens* L. (3), moderately known, often has no name, *ljutić* (spicy), *barska trava* (marsh grass) (moderately known)	It is not eaten, rarely nibbled	It was not eaten, nibbled once	In wet places in villages and marshes, leaves used as painted Easter egg pattern
*Ranunculus sceleratus* L. (2), little known, has no name, *barska trava* (marsh grass) (little known)	It is not eaten, rarely nibbled	It was not eaten	Poisonous, bloats up pigs
*Rumex crispus* L., *R. patientia* L. (1 + 1), moderately known (well known)	It is eaten if fresh	N.o	Healthy food for pigs, good against diarrhoea
*Sambucus ebulus* L. (1), well known, *apta* (well known)	It is not eaten, pigs just walk across it	It was not eaten	In open places, poisonous, used for making valuable brandy (*rakija*, sold as *alga*), jam, against fleas, deer and cattle eat
*Solanum dulcamara* L. (2), little known, *kereće grožđe* (dog grape) (little known)	It is not eaten, maybe too bitter for them	N.o	I have never tasted the fruits
*Solidago gigantea* Aiton (2), well known, name not remembered (paprat) (well kown)	It is not eaten	N.o	Grows in large patches, around marshes, a honey plant
*Sonchus arvensis* L., *S. asper* (L.) Hill. (2 + 2), well known, *‘prava’* (the real) *mlečika* (milkish), *mlečac* (milky), a kind of *boca* (spiny) (well known)	It is eaten, pigs love it, especially when young, less if too spiny, collected as fresh green fodder	N.o	It leaks milk when cut, also collected for other livestock (e.g. sheep, rabbit)
*Stellaria media* (L.) Cirillo (2), well known, *mišovkinja* (mouse grass) (well known)	It is eaten	(It was eaten/ was avoided)	In arable land, on black soil, in *Robinia* plantations, all type of livestock like it
*Symphytum officinale* L. (2), well known, name not remembered (well known)	Only the roots are eaten (leaves are not eaten)	N.o	Medicinal, nectar is a children’s snack
*Tanacetum vulgare* L. (3), well known, *smrdjlak/ smrdjlan* (smelly), a type of *paprat* (meaning a kind of weed), type of *korov* (weed) (moderately known)	It is not eaten	It was not eaten	Around marshes, but not in the water, it is smelly, poisonous, it is like a broom, rubbed on the skin against mosquitos
*Taraxacum officinale* aggr. (3), well known, *maslačak* (name refers to butter and lard) (well known)	Leaves are eaten before blooming, pigs like it very much	It was eaten several times	Milky, young leaves good for salad (but ham is a better ‘salad’…), flowers soaked in water to make honey, root is medicinal
*Trifolium pratense* L. (1), well known, *divlja detelina* (wild clover), there are 2–3 types (well known)	It is eaten when young, i.e. also in autumn, pigs like it	N.o	Doesn’t grow everywhere, veterinarians argue that it is not good for pigs
*Trifolium repens* L. (1), well known, *divlja detelina* (wild clover) (well known)	It is eaten when young, i.e. also in autumn	It was eaten several times	
*Tussilago farfara* L. (1), moderately known, *podelj, podbelj* (white below) (well known)	It is not eaten	N.o	Grows mostly on dykes and at forest edges
*Urtica dioica* L. (3), well known, *kopriva, žara* (ember, burner) (well known)	Leaves are eaten when fresh and when dry, home-kept pigs eat it more eagerly, eat roots in winter, if in need	It was eaten once	Stinky, medicinal and healthy, good for soup and salad, but ham is better…
*Verbascum lychnitis* L. (1), moderately known, name not remembered (well known)	It is not eaten, a bit if cut when young	N.o	Along roads, not in the forest
*Veronica hederifolia* L. (3), little known (little known)	It is eaten	N.o	
*Vicia sativa* L., *V. cracca* L., *V. sepium* L. (2 + 1 + 2), well known, *divlji grašak* (wild pea), *divlja grahorica* (wild faba bean) (well known)	It is eaten (It is not eaten)	N.o	
*Xanthium strumarium* L. (2), well known, *boca* (pricker), *mali čičak* (small sticking bur) (well known)	It is eaten at germination, at 2–3 leaved stage, in first 10 days, pigs love it and eat large amounts, it is not eaten later	N.o	In marshes and wet arable fields, grows after water dries up, especially in wet years, poisonous to pigs, poorly fed and hungry pigs die of it by the morning (there is no medicine against it), fruits stick to clothes, spiny fruits used by children to imitate injection needles
**Some further species**			
*Achillea* spp. (2), little known, *hajdučka trava* (outlaw`s grass) (well known)	N.d	N.o	Medicinal tea, has good smell
*Agrimonia eupatoria* L. (2), well known, *sitni čičak* (small sticking bur) (well known)	It is not eaten	N.o	Medicinal tea, sticky fruits (burs)
*Amaranthus retroflexus* L. (2), well known, *štir* (well known)	Pigs like it very much, especially young leaves and stem, also seeds, eat till full, all year round, was often collected for green fodder in the past for home and forest pigs	N.o	Grows on arable land, rare in forests
*Aristolochia clematitis* L. (1), well known, *gujina* or *vučja jabučica* (snake/wolf apple) (well known)	It is not eaten	N.o	Poisonous, not useful (not medicinal)
*Bellis perennis* L. (2), well known, *bela* (white) *rada* (female name, giving happiness)*, tratinčica?* (growing among grass) (well known)	It is not eaten	N.o	On lawns, ruminants (incl. deer) eat it
*Chelidonium majus* L. (1), little known (well known)	It is not eaten	N.o	In the village
*Clematis integrifolia* L. (1), little known (little known)	It is not eaten	N.o	In meadows, fragrant
*Colchicum autumnale* L. (0), little known, like *divlji crocus?* (wild crocus) (well known)	N.d	N.o	Doesn’t grow here [but common on Sava dykes]
*Conium maculatum* L. (1), well known, *kukuta* (well known)	It is not eaten	N.o	Poisonous, young geese and turkeys die from it
*Cuscuta* sp. (1), well known, *vilina kosa* (fairy hair) (well known)	It is not eaten	N.o	In fields, not in forests, kills clover
*Daucus carota* L. (1), moderately known, *divlja mrkva* (wild carrot), *stid cveće* (shame flower) (well known)	It is not eaten	N.o	On black earth, not in the forest, “girls lost their shame nowadays” (referring to the local name of the plant)
*Equisetum arvense* L. (1), moderately known, name not remembered (well known)	It is not eaten	N.o	In wet places, not in the forest, it has brown and green versions
*Leucanthemum ircutianum* DC. (0), moderately known, *kamilica* (chamomile) but they know it is not chamomile (well known)	It is not eaten	N.o	In meadows, rare in forests
*Matricaria discoidea* DC. (1), moderately known, *divlja kamilica* (wild chamomile) (well known)	N.d	N.o	The small one, medicinal, fragrant
*Rumex acetosa* L. (1), well known, *kiseljak* (tasting sour)(well known)	N.d	N.o	In meadows that dry up by summer, used as a children’s snack
*Solanum nigrum* L. (1), moderately known, *kereće grožđe* (dog grape), *divlje grožđe* (wild grape) (well known)	It is not eaten	N.o	In arable fields, blooms in autumn, a honey plant, maybe useful as a marihuana substitute
*Tripleurospermum inodorum* (L.) Sch. Bip. (1), well known, *konjska kamilica* (horse chamomile), *smrdljan* (smelly), *peršun* (*Petroselinum*), parsley (well known)	It is not eaten	N.o	Doesn’t grow in the forest, smelly, taller than chamomile, bittersweet (*gorčarka*), not medicinal, not good for bees
*Xanthium spinosum* L. (1), well known, *dikičina rampa, čičak* (sticky bur), *boca* (pricker) (well known)	It is not eaten	N.o	In pastures and along roads, decreasing in quantity, has painful spines

In the first column, scientific names are followed by data on frequency in the study area (4-common, 3-frequent, 2-sporadic, 1-rare, 0-missing); how well the plant is known (well, moderately, little or not known); local folk names (followed by their meaning, if known); and finally, in parentheses, the level of folk knowledge of the species in general in the Carpathian Basin (well, moderately, little or not known—based on [89, 91–93] and authors’ unpublished data)

*N.o.* no observation on foraging or avoidance of the species, *N.d.* no data

**Table 3:** Plant species (30) that were asked in interviews but were probably little or not known by local *svinjar*s (no data on pig foraging was reported or observed)

Species name	Occurrence in the area	Probable level of local knowledge by *svinjar*s	Additional information
*Acer negundo* L	Recently spreading in the area	Little known	–
*Alliaria petiolata* (M. Bieb.) Cavara and Grande	Common in shady places	Not known	–
*Alnus glutinosa* (L.) Gaertn	Probably doesn’t occur any more	Not known	–
*Anemone ranunculoides* L	Very rare in the forest	Not known	–
*Armoracia macrocarpa* (Willd.) Baumg	Rare in the marshes	Not known	–
*Asarum europaeum* L	Very rare in the forest	Very little known	Local name: kopitjnak
*Betula pendula* Roth	Probably doesn’t occur any more	Little known	Breza, in the village
*Campanula trachelium* L	Moderately rare in the forest	very little known	Local name: zvončić
*Corydalis cava* (L.) Schweigg. and Körte	Probably doesn’t occur any more	Not known	–
*Dioscorea communis* (L.) Caddick and Wilkin	Rare in the forest	Little known	–
*Frangula alnus* Mill	Not rare in the forest	Very little known	–
*Galium odoratum* (L.) Scop	Moderately rare in the forest	Not known	–
*Gentiana pneumonanthe* L	Probably doesn’t occur any more	Not known	–
*Glycyrrhiza echinata* L	Not rare in open spaces	Very little known	–
*Hottonia palustris* L	Very rare in the marshes	Not known	–
*Hydrocharis morsus-ranae* L	Common in the marshes	Very little known	A kind of lokvanj
*Lathraea squamaria* L	probably doesn’t occur any more	Not known	–
*Lathyrus vernus* (L.) Bernh	Very rare in the forest	Not known	–
*Lotus corniculatus* L	Relatively rare locally	Very little known	–
*Maianthemum bifolium* (L.) F. W. Schmidt	Probably doesn’t occur any more	Not known	–
*Melampyrum nemorosum* L	Probably doesn’t occur any more	Not known	–
*Nymphoides peltata* (S. G. Gmel.) Kuntze	Occurs on one lake only	Very little known	–
*Polygonatum multiflorum* (L.) All	Rare in the forest	Not known	–
*Ranunculus trichophyllus* Chaix	Common in marshes	Very little known	A kind of ljutić (*Ranunculus*), paprat (insignificant)
*Rhamnus cathartica* L	Not rare in the region	Very little known	Local name: kereće grožđe
*Salvinia natans* (L.) All	Not rare in flooded wetlands	Very little known	Lokvanj in the Bosut
*Silene flos-cuculi* (L.) Clairv	Sporadic in wet areas	Very little known	–
*Sparganium erectum* L	Sporadic in wetlands	Not known	–
*Stratiotes aloides* L	At only one marsh	Not known	–
*Vinca minor* L	Rare in the forest	Little known	In gardens

Ninety-eight species were reported by *svinjar*s as eaten by pigs and 56 as not eaten. There were 38 species (of the 192) for which *svinjar*s could not give information about pig foraging (how much pigs like the species). 28 species were observed by the authors as eaten regularly and 21 as nibbled by pigs, while 17 species were seen as deliberately avoided. Overlaps between *svinjar*s’ and authors’ observations were moderate. For 126 plant species authors were not able to observe any foraging or avoidance behaviour because of the lack of observed encounters with the species by pigs in the field (of the 126 species 41 species did not occur in the observation area). Leaves of 92 species, fruits or seeds of 21 species and ‘roots’ of 20 species were reported or observed as eaten.

Species that were common or moderately common in the forest but were only little known by *svinjar*s were *Circaea lutetiana*, *Moehringia trinervia*, *Veronica montana* and *V. hederifolia*, *Lysimachia nummularia*, *Glechoma hederacea*, *Brachypodium sylvaticum*, *Callitriche* sp., *Carpesium* spp. and *Genista tinctoria*. Some species were rare in the forest but were known by *svinjar*s from gardens (*Cornus mas*, *Galanthus nivalis*, *Convallaria majalis*) and *svinjar*s knew a lot about two, regionally widely known species (*Fagus sylvatica* and *Allium ursinum*) not found recently in the area. Many other of the well known species were rare in the forest but common in the arable landscapes around the villages (e.g. *Cynodon dactylon*, *Sonchus* spp.). *Svinjar*s knew many of the invasive alien species well.

For some ‘insignificant for *svinjar*s’ species, *svinjar*s did not know whether pigs eat them or not, though three were regularly observed by the authors as eaten (*Circaea lutetiana*, *Rorippa amphibia*, *Leersia oryzoides*), while others were not eaten based on our observations (*Lycopus* spp., *Carpesium* spp.). Some species were less well known by *svinjar*s than in other parts of the Carpathian Basin (*Hypericum* sp., *Colchicum autumnale*, *Achillea* sp., *Chelidonium majus*, *Lotus corniculatus*), while *Acer tataricum*, *Persicaria dubia* and the ‘small forest-*Carex’* folk taxon that includes *Carex remota*, *C. sylvatica* and *C. divulsa* were better known.

The most common plant forages of pigs (mentioned or observed) were fruits of trees (esp. *Quercus* and fleshy forest fruits), marsh grasses (esp. *Agrostis* and *Glyceria*), forest herbs (esp. *Ranunculus ficaria* and *Circaea lutetiana*), marsh plants with nutritious ‘roots’ (esp. *Carex* spp. and *Iris pseudacorus*), shrubs and young trees with young fresh leaves (esp. *Crataegus* and *Carpinus*), as well as ‘tame’ plants that grow in the sun (“*pitomina”*, e.g. *Persicaria dubia* and *Erigeron annuus*). Some species were reported as especially loved by pigs (even if some were less available to them): *Quercus robur* acorns (it is the primary forage, always eaten if available), *Polygonum aviculare*, *Amaranthus retroflexus* and *Sonchus* spp. leaves, and *Pyrus* sp., *Juglans regia*, *Prunus cerasifera* and *Trapa natans* fruits. *Svinjar*s spoke enthusiastically about how eagerly pigs forage on these species.

Some species (e.g. *Salix alba*, *Ulmus minor*) were not eaten or only rarely because in April-June many other—“*sweeter*”—species were available (“*nothing forces pigs to eat them, pigs are choosy in these months*”). *Svinjar*s reported that pigs graze more grass in wet years, and even eat grass from below the water. Many of the invasive alien species were not eaten by pigs, e.g. *Ambrosia artemisiifolia*, *Amorpha fruticosa*, *Asclepias syriaca*, *Phytolacca americana*, *Robinia pseudoacacia*, *Solidago gigantea*, *Sorghum halepense*, *Vitis* sp., while *Erigeron annuus* (little known by *svinjar*s) was regularly eaten.

*Svinjar*s argued that traditional pig breeds (e.g. the black coloured Sremska Lasa breed) were less selective when grazing and more ‘knowledgeable’ about edible forest and marsh plants, as they received less additional fodder (corn).

Foraging varied greatly depending on the season. Pigs grazed on marsh and forest plants and anything that remained green in winter while foraging dominantly on *Quercus robur* acorns and/or earthworms. Pigs loved the young fresh herb, grass and shrub leaves (especially *Agrostis stolonifera*, *Ranunculus ficaria*, *Crataegus* spp.) sprouting in spring, and foraged on the remaining acorns. By mid-summer leaves hardened or dried: “*the forest is empty, hungry*”. Pigs grazed in sunny places and on grasses and waterweeds of drying and waterlogged marshes. *Svinjar*s recalled that in the past, pigs were driven to feed on the weeds and crop residues of stubble fields in this “*empty*” period. In August and September acorns start falling, and forest fruits (cherry plum in July, followed later by wild pear and apple) start ripening. Pigs foraged on acorns (loved more after rains) and on fallen tree leaves (mostly *Acer campestre* and *Fraxinus angustifolia*). Foraging during the autumn and winter season depended on the availability of acorns. In snowy winters, pigs foraged more on the ‘roots’ of marsh plants (e.g. chewing on *Carex* rhizomes) and, when in severe need, on certain wild fruits (*Prunus spinosa*, *Crataegus* spp.). If there were no acorns, they searched for earthworms and ate remaining fruits and green leaves. Pigs ate’roots’ (but only of non-woody species) mostly in winter, and especially in forage-poor years.

Contradictory information on foraging was rare. *Svinjar*s disagreed on altogether 8 species, but three were rare in the forest (*Viola alba*, *Marsilea quadrifolia*, *Typha* spp.); in four cases (*Viola reichenbachiana*, *Oenanthe aquatica*, *Plantago major* and *Primula acaulis*) one or two *svinjar*s disagreed with the others whether it was foraged or not. *Svinjar*s’ reports contradicted our observations only for *Erigeron annuus* and *Glechoma hederacea* and for a further 5 species, but for the latter the difference was only whether the species were ‘not eaten’ or ‘only nibbled’ (see Table 2 for details).

Some plants were foraged only in a short time-window and/or in a given phenological state (e.g. young leaves of tree and shrub species mainly in the spring, *Juncus* ‘roots’ in snowy winters). Most plant species were foraged only for very short time periods during the observations (less than a minute even if available), except: acorns, marsh grasses (*Agrostis*, *Glyceria*), *Carex* rhizomes, *Polygonum aviculare*, *Ranunculus ficaria*, *Crataegus* spp. leaves, *Lemna-Spirodela* spp., *Glechoma hederacea*, *Circaea lutetiana*, and fallen leaves of *Acer campestre* and *Fraxinus angustifolia*.

Some species were reported as only eaten by pigs if nothing else was available (famine foods for pigs). For example, the fruits of *Acer campestre*, *Rosa canina* and *Cornus sanguinea*, and the ‘roots’ of *Carex elata*, *Juncus effusus* and *Urtica dioica*. *Svinjar*s added: “*They have to love them… if they are in need* [food shortage].” Some plant species were reported as being eaten differently by or having a different impact on pigs (of the same breed), depending on whether they were kept at home in the village or in the forest (*Chenopodium album*, *Urtica dioica*).

Grasses and herbs growing in sunny places at forest edges, along lanes and roadsides were called “*pitomina*” (from the word tame and animal feed), as opposed to the lower quality grasses and herbs growing in the shade of the forest. *Persicaria dubia* (*P. mite*) was reported and observed as being mostly eaten in sunny places. Pigs grazed ‘precisely’, i.e. they were able to focus on the preferred species. For example, they grazed *Rorippa amphibia* from among *Mentha aquatica*, and *Glyceria* and *Agrostis* grasses from among *Chaiturus (Leonurus) marrubiastrum*.

*Svinjar*s rarely drove pigs deliberately to forage on specific species and in specific places, and did so mostly in the past: *Quercus robur* and *Q. cerris* acorns, grassy village pastures in summer, beech mast (to mountains, but only in the past), waterweeds (*lokvanj*) in the rivers, and stubble fields. Driving and keeping pigs away from certain forages or sites was still practised: unharvested arable crops, reforestations (with acorns), areas designated for acorn collection, hunting areas, and to prevent damage to river dykes, railway and road verges.

*Svinjar*s argued that “*those pigs are the most ‘beautiful’ which can go wherever they want.*” “*The 12-year-old sows know exactly where to look for what they want*.” “*Pigs know the forest six times better than me, although I also know each and every tree…*” *Svinjar*s were aware of individual and herd-level differences: “*You know, some of my pigs eat mushrooms, others don’t, they learn which mushrooms are edible from other herd members*.” We also observed that *Ranunculus ficaria* was eaten often by one herd and much less often by the neighbouring herd.

### Knowledge generation about plants and pig behaviour

When asked about their knowledge of the forest, plants and pigs, and life in the forest, *svinjar*s argued that “*I was born into this forest*.” “*As if we were ‘shot’ into the forest.*” “*Here everything is clear to me*.” The main source of knowledge was direct observation of pigs (see answers in Table 2). *Svinjar*s were observant people. “*Pigs mostly eat it* [*Crataegus* spp. and *Prunus spinosa* fruits, *Juncus roots*] *in winter if there is snow*.” “*This plant* [*Lamium galeobdolon*] *doesn’t grow in the forests I went to with my pigs*.” “*I saw this plant* [*Myosotis scorpioides*] *in her mouth but did not actually see it being swallowed*.” “*I have never seen pigs bother with žesta* [*Acer tataricum*].” “*I love watching birds and other animals*.” “*I watched it *[*Dryopteris*] *for years*, [I am sure] *it has no flowers*!”

On the other hand, *svinjar*s lacked knowledge about certain plants. “*I am used to seeing these plants*, [but] *I do not observe them carefully*.” “*These* [two plants: *Carex remota* and *C. divulsa*] *are the same to me, but I know that they are not the same to you.*” Plants regarded as useless or not important were called “*korov*” (weed) or “*paprat*” (i.e. fern but meaning ca. weed/insignificant), and many Asteraceae species were called “*kamilica*” (chamomile) or “*divlja* [wild] *kamilica*” though *svinjar*s knew that it was not the real chamomile.

*Svinjar*s regularly but not often followed their free ranging pigs into the forest, even less often into marshes. “*Pigs like freedom, like we do. They know where the best acorn is*.” However, they did keep an eye on how far pigs went, and whether pigs entered into forbidden areas (forests closed from grazing, road verges and arable fields). Over many decades, *svinjar*s accumulated a large amount of experience about plants and pigs.

The interviewed *svinjar*s had decades-long and personal experience with forest pigs. “*Till it goes ‘through your back’, you don’t know it*.” (i.e. effective learning needs personal experience). “*You have to see them every day from their birth to know them well*.” And they do visit pigs almost every day, all year round, bringing food and checking on health and piglets. *Svinjar*s spoke from experience, having lived through forage-rich and forage-poor years and seasons, and observed rare events (floods, severe winters) and long-term gradual changes of their environment. For example, the long-term impact of river regulation on the hydrology and species composition of marshes and forests, the impact of cattle grazing on the forests and marshes and the consequences of the abandonment of cattle and sheep grazing, the impact of pollution on waterweeds in the Bosut river, and the spread of new (invasive alien) species. *Svinjar*s did not guess an answer to our questions, instead they said: “*I don’t know whether pigs eat it or not*.” “*I don’t want to say stupid things when I simply don’t know this*.” Or simply closed the conversation by saying: “*You know, everything is connected in the world*.” “*Nature is a wonder both to us and to you…*”.

Some *svinjar*s were eager to learn from parents, grandparents, other old villagers and from respected foresters. *Svinjar*s regularly recalled stories elders had told them (Table 2, e.g. why *Ajuga reptans* is green in winter, the incredible endurance of *Elytrigia* and *Cynodon* rhizomes, masting in far-away mountains in the past). Other *svinjar*s claimed: “*I was never interested in what grandpa showed us*.” But all agreed: “*Those old guys knew more* [about pigs, forests and plants] *than we do*.” *Svinjar*s regularly visited their neighbour *svinjar*s in the forest and shared information on pig movements (e.g. lost males), currently available forages, the impact of recent weather events (e.g. heat days on acorns), and changing forestry and veterinary regulations. Most local plant names documented in Table 2 were widely shared among *svinjar*s.

All *svinjar*s also had work experience as farmers, and most had also been employed as forest workers. In their childhood they played a lot in the forest, collected edible and medicinal plants, mushrooms, flowers, fodder for the pigs (acorn, *Chenopodium*, *Amaranthus* and *Elytrigia* from arable fields), and wood for firewood, huts, tools and sticks. They had therefore been familiar with many (probably most) plant species since early childhood. *Svinjar*s remembered some practices from their childhood when they pastured pigs on weeds in stubble fields. They also acquired plant knowledge from foresters. They often recalled Dr. Josip Erdeši, former head of the Višnjićevo forestry office, who respected *svinjar*s and supported pig grazing in the forests (Erdeši said to *svinjar*s: “*as long as the livestock is in the forest, it is beneficial to the forest, and when there is no more livestock in the forest, the forest will become ill*”). TV and school were rarely mentioned as sources of plant knowledge (except in connection with the marketed wild green *Allium ursinum*). During the years of our research, *svinjar*s became more interested in some plant species and the nuances of foraging behaviour. “[Last year] *I told you that pigs don’t eat this* [*Lysimachia nummularia*] *but now I can see they are eating it*.”

## Discussion

### Knowledge of plants and foraging pigs

*Svinjar*s distinguished between at least 181 wild plant taxa in the studied forested floodplain, and had knowledge of 154 species foraged regularly or rarely, or avoided by pigs. This is the highest number of species documented for foraging domestic pigs in Europe (cf. [25, 26, 37]). The depth of ethnobotanical knowledge was comparable to other Central European traditional ecological knowledge-rich regions (e.g. Gyimes in Romania [91]; Hortobágy in Hungary [89], and also comparable to the length of lists of plant species foraged by wild boar [64, 69, 70].

*Svinjar*s saw and understood many forest and marsh plant species “through the mouth” of their pigs (loved, nibbled, avoided, toxic, medicinal, cf. [51]) but were also knowledgeable about the ecological needs (e.g. habitat requirements) and human uses (medicinal, tools, wild food etc.) of the species. *Svinjar*s had especially rich knowledge on some plant species (and groups), for example on *Quercus robur* and its acorns, *Ranunculus ficaria*, marsh grasses, *Carex* spp. For these species, foraging behaviour was reported in a nuanced way.

The low number of interviewed informants (however knowledgeable) is an obvious limitation of our study (cf. [94]). We were able to reach the saturation of information only for the well and moderately known species. Some plants, despite being common in our study area and even eaten by pigs, were little known by *svinjar*s. A possible reason for uncertainties in the knowledge of *svinjar*s may be their lack of awareness of some ‘insignificant’ plant species (e.g. *Circaea lutetiana*, *Veronica montana*, *Glechoma hederacea*, *Ranunculus trichophyllus*, *Teucrium scordium*, *Stachys palustris*). Such ‘ignorance’ was also found in other ethnobotanical studies in the region [89, 91]. Furthermore, *svinjar*s were cautious and tried to base their answers on their own observations. They all knew that we are botanists and have detailed knowledge of the local flora and already have some observational experience on pig foraging, so were careful with their answers. Some gaps in *svinjar*s’ knowledge may be due to a lack of knowledge (these plants were never known well in this area) or recent loss of knowledge (ancestors knew the species but it is not any more known) (cf. [95]).

Our own observational research also failed to produce a full picture of pig foraging. For many species we lacked visual observation data because (1) areas where these species grow were not utilized any more by pigs (stubble fields, old-fields, grassy pastures, road verges in arable land); (2) some species were rare in the forests or did not occur in the areas where the observations were done; and (3) some species are eaten only rarely and/or in a narrow seasonal window and/or under specific weather conditions (snow) or as a famine food, hence foraging was not observed.

Studies on pig foraging in open landscapes have found that herbaceous species are a staple food for pigs [23, 25, 26]. This was also the case in our research. The year-round main forages of pigs were similar to those of wild boar (grasses and herbs in spring, crops in summer, forest fruits and acorns in autumn, and acorns, grasses and roots in winter [64, 65] and references therein) except for crops. As we also observed in the case of pigs, wild boar prefer grasses over forbs [65, 96], but see the opposite for pigs in Von Flegler et al. [25].

The only available published comprehensive list of plant species foraged by free-ranging domestic pigs was compiled by Von Flegler et al. [25]. Their list shares many similarities with our list, although they studied grassland pastures and not forests (pigs love *Cirsium arvense*, *Taraxacum officinale*, eat regularly *Agrostis stolonifera*, *Bidens frondosa*, *Iris pseudacorus*, *Stellaria media*). Wild boars also forage on many wild herbaceous species, though usually there is no quantitative information available: *Taraxacum officinale*, *Convolvulus arvensis*, *Moehringia trinervia*, *Stellaria media*, *Rumex acetosa*, *Pulmonaria officinalis*, *Cynodon dactylon*, *Symphytum officinale*, *Trifolium* spp., *Urtica* spp. [64, 69, 70].

Historical-ethnographic sources on traditional pig keeping from the region list relatively few wild plant species eaten by pigs (see [32] and Öllerer et al. ined.), probably because ethnographers and historians were interested in the extensive land-use practices in general and focused especially on the social background and social organization, and on the use of agricultural tools and buildings for livestock. We can only presume that Central European ethnographers and local historians did not use the free-listing method as a way of elicitation, as only the most salient species (e.g. *Quercus* spp., *Typha*, *Phragmites*) were listed in the publications. None of the ethnographic-historical sources mentioned the regularly eaten (and loved) *Amaranthus retroflexus*, *Polygonum aviculare*, *Chenopodium album*, *Convolvulus arvensis*, *Trifolium* spp. and *Taraxacum officinale* as foraged. Studies on wild food plants may also list species eaten by (given to) pigs (e.g. [97]).

Acorns were a staple forage, and *svinjar*s kept estimating the potential autumn yield from April onwards. This was a widespread practice in the past throughout Europe [20]. Only fallen acorns were eaten in our study area, *svinjar*s did not shake or beat down acorns with sticks in August–September, a practice known in medieval times [14] and still applied by local *svinjar*s as recently as 30–40 years ago.

Pigs in the Bosut forest loved fleshy forest fruits. Wild boars also often feed on almost all fruits available in their territory (2–3% in volume [64]). On the Drava floodplain, Tucak [70] documented foraging by wild boar on *Prunus domestica*, *Morus alba*, *Corylus avellana*, *Rubus* sp., a similar list to ours. Most cultivated fruits were given traditionally to Iberian pigs as supplementary fodder [63]. Fruit consumption is also important ecologically as it contributes to the dispersal of these species in the forested landscape [98].

We found 20 species whose ‘roots’ were foraged by pigs. Pigs studied in Germany foraged on the roots of many herbaceous species (e.g. various grasses, *Cirsium arvense*, *Taraxacum officinale* and *Urtica dioica* [25], also observed by Stolba and Wood-Gush [23]), while dehesa pigs eat much fewer roots (< 1% in volume [28]). Ethnographic sources also often mention digging for ‘roots’ of wetland plants (mostly but not exclusively in winter and early spring) [12, 15, 99, 100]. Roots of marsh plants can also compose the bulk of food for wild boars living in wetlands, although it remains a second-choice food source [101].

Literature sources (both historical, e.g. [99, 102], and recent, e.g. [23] mention that pigs forage on the roots of woody forest species, but the species are rarely documented (for wild boar, e.g. [69, 70]). We tried to document this behaviour, but not once did we observe pigs foraging on the roots of woody species, and *svinjar*s were also convinced that it did not take place (“*domestic pigs do not need these roots, they never eat woody roots, they get corn from us*”). Roots were only broken and torn, or if pigs did take them into their mouths, they ultimately discarded them. In the Drava floodplain, wild boars consumed, in the four seasons from spring to winter, 20%, 9%, 15% and 48% roots, respectively (in volume [70], but woody and non-woody species were not distinguished), including roots of *Quercus robur*, *Populus* spp., *Salix* spp., *Corylus avellana*, *Acer* spp., *Robinia pseudoacacia*, as well as of non-woody species such as *Symphytum officinale* (most commonly in winter and spring), *Taraxacum officinale*, *Trifolium* sp., *Pteridium aquilinum* and grasses [70]. We did not observe pigs foraging on grass roots in our study area at any time.

There is almost no data on browsing by wild boar and little by pigs. *Svinjar*s argued that pigs prefer leaves over twigs, the opposite of red deer, and we also observed grazing much more often than browsing. Wild boars also rarely browse [65, 103, 104], max. up to 5% in volume [105]. Browsing by pigs is rarely mentioned in recent (Von Flegler et al. 2005, 2.6%) or historical sources (on *Fagus*, *Carpinus*, *Fraxinus*, *Ulmus*, *Acer* and *Salix caprea* [106], and *Crataegus* sp. [107].

In spite of domestication, the behaviour of domestic pigs greatly resembled that of wild boar (cf. [23]). Consequently, the direct visual investigation of these easily observable domestic pigs could provide a reasonable basis for formulating research hypotheses for studies on the foraging behaviour of wild boar or for a better understanding of causes shaping wild boar grazing and rooting patterns (cf. [108]).

### Knowledge generation about pigs and plants

*Svinjar*s were born and embedded into this lifestyle, were knowledgeable about plants and pig foraging, and emphasized the importance of personal, long-term, everyday experience with the animals and the forest. This understanding of how adequate knowledge of animal keeping and pastures is generated is universal among herder and pastoralist communities [89, 109, 110].

Though *svinjar*s spent a lot of time with their pigs in the forest, they usually did not follow them on their foraging trips. This may be one of the reasons why some species and their consumption remained outside the knowledge of the *svinjar*s. Inga [111] found that the knowledge of Sami reindeer herders about summer forages was significantly lower than about winter (e.g. lichen) forage, because the interviewed Sami herders usually did not follow their reindeers during summer months. By contrast, many European sheep and cattle herders still closely follow their herds during the whole grazing season, develop a deep understanding of foraging preferences and behaviour, and even use this knowledge to moderate appetite and increase forage intake [5, 52].

We found similar patterns and ways of ecological knowledge generation and knowledge transmission to those documented in other traditional indigenous and local communities [112–114]: vertical learning (within genealogical lines), oblique knowledge transmission (between genealogical lines—e.g. older colleagues) and horizontal transmission (between members of the same generation). The dominance of shared folk plant names was an indication of the operation of knowledge transmission mechanisms and indicated frequent contact and interaction between *svinjar*s [115]. Personal experience with nature completed knowledge transmission, while non-local sources, information gained from school or media (books, newspapers, TV, internet), were rarely mentioned, as information on pig foraging is probably rare in these sources (cf. [89]). Based on our limited number of data, personal observation seemed to be more important with regard to forage-related traditional knowledge than vertical learning from older community members (cf. [116]).

Our research definitely had an impact on knowledge generation: *svinjar*s became more observant on some species. This is also a general phenomenon, as herders like to learn from people whom they consider, in their own judgement, to have reliable and relevant knowledge [5].

Traditional knowledge on forages and foraging was eroding in the study area, while knowledge of particular species is becoming less in-depth (cf. [95]). Similarly to other local knowledge holders worldwide, the interviewed *svinjar*s argued that they knew less about the plants than their fathers and grandfathers did. *Svinjar*s also emphasized the diminished dependence on pigs and local natural resources, which led to less effective knowledge generation (they accompany their pigs less often than in the past). Formal education, connections to the market economy and changing lifestyles may reduce not only the amount of local ecological knowledge but also the willingness of younger generations to continue this lifestyle (cf. [117]). The almost complete absence of the next generation of *svinjar*s has practically ended knowledge transmission. The decreasing number of practising *svinjar*s may also have caused a decline in the diversity of experiences. The ageing and dwindling community of *svinjar*s is leading to the disappearance of the informal socio-cultural institutions of knowledge transmission and eliminating opportunities for imitation and improvisation as important mechanisms in knowledge acquisition [114, 118, 119].

*Svinjar*s were eager to teach us their knowledge and hoped that science could help archive their knowledge, and even improve knowledge transmission by raising interest among the younger generation to learn more about traditional pig keeping [a new local association of pig keepers was recently formed to preserve traditional farming practices (*svinjar*s’ pers. comm.)]. However, economic drivers, the worsening of the local and the national regulatory environment and the spread of modern lifestyles still threaten the continuation of this traditional forest-marsh pig keeping practice [31, 32].

### Future potential of this almost lost traditional practice

The historical practice of extensive pig grazing is regularly advocated for use in land management, for example, in forestry [34, 102, 120, 121], nature conservation management [6, 21, 40, 41], and organic/environmentally friendly farming [34, 38, 39].

The possibility of using pig grazing and rooting in nature conservation management is, however, hardly understood [122], and may also be misunderstood and undervalued [41]. Evidence shows that extensive pig keeping is an efficient way of managing some mud specialist species and of restoring formerly grazed marshes [6, 40]. For example, several red-listed marsh plant species (*Marsilea quadrifolia*, *Ludwigia palustris*, *Elatine* spp., *Hottonia plaustrisi Lindernia procumbens* etc. [6]) benefit from pig grazing, as do some birds (waders, ducks and geese [41]). Pigs were also suggested for managing wood-pastures and open forests, based on their interwoven history [14] and on the experience of the ongoing practice in the dehesas (e.g. [27]).

The carefully controlled application of livestock grazing in certain forest management situations is recommended by Öllerer et al. [46], which is in line with the knowledge and view of some forestry experts (e.g. [84, 123–125]). Data on the forestry use of pig grazing are, however, also scarce. Historical data indicate that pigs were used before the planting of tree seedlings to eat up wormy, unhealthy acorns and to loosen up the soil, mixing it with leaf litter in order to achieve better conditions for the germination of healthy acorns that fell subsequently [85], but also to provide protection against *Rubus* and weed invasion and against pests (insects, small rodents) [120, 121, 124–126]. However, more research is needed to understand the potential benefits of pig grazing in present-day forestry systems, as habitats (their hydrology, abundance of invasive species) may have changed considerably since the nineteenth century [127]. The use of free-ranging pigs in modern agroforestry systems (e.g. with fast growing tree crops [38, 39] is a novel practice of raising pigs in an environmentally friendly way. The use of pigs to open up dense encroached secondary forests to more intensive use and to loosen and clear their soil for agriculture is being tested on US and UK farms [128, 129].

Knowing the often detrimental impact of wild boars on semi-natural habitats [130] one may ask: are domestic pigs better than wild boars? We cannot answer this question for certain at present (there are no comparative studies in which spatial and temporal constraints etc. are controlled for), but we can be sure that domestic pigs seem to forage somewhat differently, while their impact can be managed more easily in space and time (to protect sensitive semi-natural habitats and crop fields). Historical data show that domestic pigs were not listed among the livestock types harmful for forests and the pig was the last livestock type excluded from the forests in the region [131].

We made our study in a high conservation value floodplain area that desperately needs an improved conservation management system. Floodplains are dynamic systems, where extreme events (especially unpredictable major floods) challenge conservation and the utilization of natural resources [29]. Innovations and creativity are needed for sustainable management and conservation. The studies of Kiš et al. [31, 32] show that extensive pig grazing has a place and role in this, especially with regard to maintaining open habitats and increasing water infiltration. Local pig keeping methods develop in order to adapt to market changes and new forestry and veterinary regulations, while still preserving many ancient elements. The adaptive capacity of the system is, however, limited, and needs strengthening.

We do not know what knowledge humanity will need in the coming decades and centuries in order to develop and survive. Scientists warn that ancient knowledge needs recognition and support at multiple levels in order to continue adapting [2, 3]. Consequently, national and local governments, veterinarians, forestry companies and nature conservation institutions all have a share of responsibility in maintaining this special pig keeping practice for the future.

## Conclusions and outlook

Traditional pig keepers (*svinjar*s) in Serbia developed their deep ecological knowledge of plants and foraging behaviour of pigs by maintaining a close relationship with the forest, the pigs, plants and wild animals, and the knowledge of their ancestors. We started to document this huge body of knowledge, which is vanishing rapidly because of internal (e.g. livelihood changes) and external (e.g. markets, regulations) drivers.

A much deeper scientific understanding is needed of this traditional pig grazing practice to efficiently harness its potential for conservation management in protected areas and for organic farming (and forestry), and to understand its past impacts on the ecology of European deciduous forests. Knowledge co-production with further scientific disciplines and professions (e.g. ethnozoology, animal behaviour science, ecology, veterinary and food science) is needed to cover the wide spectrum of *svinjar*s’ knowledge. *Svinjar*s with their pigs convert inedible (i.e. not consumed by humans nowadays) biomass, such as forest and marsh grasses and herbs, acorns, roots and earthworms, into high quality meat, using little modern technology. This knowledge is an invaluable intangible cultural heritage of Serbia.

Only a few *svinjar*s remain in the Bosut floodplain, and hardly any of them is under the age of 60. If external drivers are not managed properly, this practice may disappear very soon. Decision makers, in particular forestry, nature conservation, hydroengineering and veterinary experts and officials, need to recognize *svinjar*s’ knowledge and practices, support ongoing and future development and adaptation of this traditional practice, and support bottom-up initiatives to develop and promote local products and tourism. We hope that our study will encourage others to delve deeper into the knowledge and methods of traditional pig keeping in forests and marshes, and help the survival of this ancient practice.

## Data Availability

Databases used and cited are available upon request at the Institute of Ecology and Botany, Centre for Ecological Research, Vácrátót, Hungary.
